# General health, anxiety, and depression: relationship with perceived self-efficacy and positive mental health in nursing students*

**DOI:** 10.1590/1980-220X-REEUSP-2025-0260en

**Published:** 2025-11-17

**Authors:** Eliel de Oliveira Bandeira, Daniele Alcalá Pompeo, Natália Sperli Geraldes Marin dos Santos Sasaki, Camila Garcel Pancote, Luiz Antônio Alves de Menezes-Júnior, Danilo de Miranda Alves, Letícia Palota Eid, Luciano Garcia Lourenção

**Affiliations:** 1 Universidade Federal do Rio Grande, Escola de Enfermagem, Rio Grande, RS, Brazil.; 2 Faculdade de Medicina de São José do Rio Preto, São José do Rio Preto, SP, Brazil.; 3 União das Faculdades dos Grandes Lagos, São José do Rio Preto, SP, Brazil.; 4 Universidade Federal de Ouro Preto, Escola de Nutrição, Ouro Preto, MG, Brazil.; 5 Universidade Federal de Jataí, Instituto de Ciências da Saúde, Jataí, GO, Brazil.

**Keywords:** Students, Nursing, Mental Health, Self Efficacy, Anxiety, Depression

## Abstract

**Objective:**

To analyze the general health, anxiety, and depression of nursing students and analyze their relationship with the perception of general self-efficacy and positive mental health while coping with COVID-19.

**Method:**

A cross-sectional, descriptive, and correlational study with 138 nursing students from a public institution. Instruments were applied for sociodemographic variables, the Hospital Anxiety and Depression Scale, the General Self-Efficacy Scale, the General Health Questionnaire, and the Positive Mental Health Questionnaire. The analyses included descriptive statistics, correlations, and univariate and multivariate logistic regression.

**Results:**

A 5% increase in the Positive Mental Health score was associated with a 20.4% reduction in the probability of anxiety (OR = 0.83; 95% CI: 0.73–0.93) and a 26.6% reduction in the probability of depression (OR = 0.79; 95% CI: 0.70–0.89). Similar increases in the General Health Questionnaire were associated with higher probabilities of anxiety (29%) and depression (18%). A 5% increase in the perception of general self-efficacy reduced the probability of both outcomes (anxiety and depression) by 12.4%.

**Conclusion:**

Positive mental health and self-efficacy were protective factors, reinforcing the importance of institutional actions to promote academic well-being.

## INTRODUCTION

The transition to university life represents a critical stage in the development of young adults, marked by significant changes in the teaching-learning model, social interactions, and emotional structure^(1)^. For health students, such as nursing students, this adaptation also involves experiencing emotionally demanding situations and high responsibility, which can compromise psychological well-being and increase vulnerability to the development of mental disorders, such as anxiety and depression^(2)^.

Several studies indicate that nursing students experience higher levels of psychological distress compared to their peers in other courses, due to the extensive workload, the intensity of practical activities, and early exposure to situations of pain, suffering, and death^(3)^. These conditions make this group especially susceptible to mental illness, with negative implications for academic performance, the risk of dropout, and the quality of professional training^(2,4)^.

The COVID-19 pandemic has exacerbated this scenario by imposing social isolation measures and abrupt changes in the teaching-learning process, with the migration to remote learning and the suspension of practical activities essential for nursing training^(5)^. Research has reported a significant increase in symptoms of anxiety, stress, and depression among university students, especially those in the health field, who have come to be recognized as a priority group for mental health promotion and care actions^(6,7)^.

In this context, self-efficacy, defined as the belief in the ability to organize and execute actions to achieve certain goals, has stood out as a protective factor for the mental health of university students^(8)^. Individuals with high levels of self-efficacy tend to view academic challenges as opportunities for growth, exhibiting greater emotional resilience, less reactivity to stress, and less propensity for anxiety and depression^(9)^.

Positive Mental Health (PMH), in turn, represents an approach focused on psychological well-being that goes beyond the mere absence of mental illness. It involves aspects such as personal satisfaction, self-control, relationship skills, autonomy, and personal fulfillment^(10)^. The literature has emphasized the importance of promoting it from the earliest stages of academic training as a strategy to strengthen social-emotional skills and prevent psychological distress^(11)^.

Despite advances in scientific research on the mental health of university students, there are still few studies that analyze, in an integrated manner, the links between general health, emotional symptoms (anxiety and depression), perceived self-efficacy, and positive mental health, especially among nursing students in Brazil. This gap is even more evident given the disproportionate impact of the pandemic on this group and the need to generate evidence to support institutional actions to welcome, prevent, and promote university well-being^(6)^.

This study seeks to expand the literature by examining, in an integrated manner, variables traditionally analyzed in isolation, such as positive mental health, perceived self-efficacy, anxiety, depression, and general health, contributing to a more comprehensive understanding of the mental health of nursing students. The starting point is the hypothesis that higher levels of positive mental health and self-efficacy are associated with lower levels of anxiety and depression, while poorer general health conditions are related to higher risks of emotional distress.

Given the above, the study aimed to analyze the general health conditions, anxiety, and depression of nursing students and analyze their relationship with the perception of general self-efficacy and positive mental health while coping with COVID-19.

## METHOD

### Study Design

This is a cross-sectional, descriptive, and correlational study conducted with nursing students from a public higher education institution in the interior of the state of São Paulo, Brazil. The manuscript was structured according to the guidelines of the STROBE (Strengthening the Reporting of Observational Studies in Epidemiology) tool.

### Study Location

The study was conducted at the São José do Rio Preto School of Medicine (Famerp), which offers undergraduate courses in Medicine, Nursing, and Psychology, as well as Lato Sensu and Stricto Sensu graduate courses. The institution was chosen because of its relevance in training health professionals and the increased demand for psychological support services among its students, observed especially during the COVID-19 pandemic.

### Population, Selection Criteria, and Sample

The study population included 190 students enrolled in the Nursing course in 2020. Participants were identified using a list provided by the educational institution. The inclusion criteria were: being regularly enrolled in the institution’s undergraduate nursing program; being ≥ 18 years of age; and being present on the data collection dates.

For the sample calculation, a confidence level of 95%, a margin of error of 5%, and an expected proportion of 50% were considered, resulting in an estimated minimum sample of 128 students.

A non-probabilistic convenience sampling was adopted, including all students who consented to participate and fully responded to the instruments. A total of 138 students participated in the study, representing 72.6% of the total population, which ensures an adequate proportion for the study objectives.

### Data Collection

Five self-administered instruments were used for data collection: a questionnaire containing personal, social, and academic variables, lifestyle habits, as well as questions about the presence of stress, overload, and perception of the impact of the pandemic on their lives; the Hospital Anxiety and Depression Scale (HADS)^(12)^; the General and Perceived Self-Efficacy Scale (Escala de Autoeficácia Geral e Percebida)^(13)^; the General Health Questionnaire (Questionário de Saúde Geral - QSG-12)^(14)^; and the Positive Mental Health Questionnaire (Questionário de Saúde Mental Positiva - QSMP)^(15)^.

The HADS measures symptoms suggestive of anxiety and depression in non-psychiatric settings, based on the individual’s last week. Each item on the HADS has four possible responses (0 to 3), for a maximum score of 21 points for each subscale^(12)^. Students with a score ≥ 9 on each subscale were considered to have anxiety and/or depression.

The General and Perceived Self-Efficacy Scale consists of 10 items, with responses ranging from one to five, where 1 means I totally disagree and 5 means I totally agree. Each item measures the individual’s beliefs about their ability to achieve a goal, deal with a situation, or perform a task (Example: I can always solve difficult problems if I try hard enough; it is easy for me to stick to my goals and achieve my objectives). The score is calculated by adding up the values of the items, with a higher score indicating a greater perception of self-efficacy, on a scale of 10 to 50^(13)^.

The QSG-12 is an abbreviated version of the General Health Questionnaire, which was initially developed with 60 items. The 12-item version is the shortest and most widely used to measure psychological well-being, as evidence has shown that reducing the number of items does not compromise the reliability of the responses. The QSG-12 consists of 12 items that assess how much the person has experienced the symptoms described, with four possible responses. For items that deny mental health, the response options can be: (1) absolutely not, (2) no more than usual, (3) a little more than usual, and (4) much more than usual. For positive items, the responses range from: (1) more than usual, (2) the same as usual, (3) less than usual, (4) much less than usual. It should be noted that the negative items were reversed and the lowest score indicates a better level of well-being^(14,16)^. In Brazil, the QGS-12 has demonstrated evidence of validity and accuracy, as well as internal consistency, evaluated by Cronbach’s alpha above 0.80^(17)^.

The Positive Mental Health Questionnaire (QSMP) measures overall positive mental health through 39 statements about each individual’s way of thinking, feeling, and acting^(15)^. These statements were grouped into six factors: (F1) personal satisfaction, (F2) positive attitude, (F3) self-control, (F4) autonomy, (F5) personal fulfillment and problem solving, and (F6) interpersonal skills. Responses to each item are presented on a 4-point Likert scale: (1) always or almost always, (2) most of the time, (3) sometimes, (4) almost never or never. Some items in the instrument are described in a positive way (n = 19) and others in a negative way (n = 20), in an attempt to identify possible flaws in the completion, such as automatic marking of items. QSMP scores range from 39 (minimum score) to 156 (maximum score). The higher the score obtained, the higher the level of positive mental health^(15)^. The Portuguese version was validated in a sample of 942 university students, with good internal consistency results (Cronbach’s alpha = 0.92) and a factorial structure similar to the original study^(15)^.

The data were collected in the second half of 2020. The invitation to participate in the study was sent to all nursing students, to the email address provided at the time of enrollment and made available by the institution, and by WhatsApp message to the undergraduate class groups. The invitation consisted of an informative video that presented the title of the research, its objectives, those responsible for conducting it, and the instruments that should be answered, in addition to emphasizing that participation was voluntary and that the study complied with ethical recommendations for studies involving human subjects.

Upon agreeing to participate in the study, students clicked on a link provided at the end of the video, which directed them to the Free and Informed Consent Form (FICF). After reading it, students had the option to agree to participate in the study. After confirming their acceptance, students had access to the data collection instruments (sociodemographic questionnaire, HADS, General and Perceived Self-Efficacy Scale, QSG-12, and QSMP, all made available through the Google Forms platform. The use of this tool was essential for data collection, due to the study being conducted during the period of social isolation resulting from the health situation generated by the COVID-19 pandemic, in which students were away from on-site activities.

### Data Analysis and Processing

Statistical analysis was performed using Stata® 15.0 software, adopting a significance level of 5%. Initially, descriptive analyses (frequencies, means, and standard deviations) and bivariate tests (Pearson’s chi-square and Student’s t-test) were performed to compare anxiety and depression outcomes with sociodemographic variables. The correlation between the HADS, QSG-12, Self-Efficacy Scale, and QSMP scores was assessed using Spearman’s coefficient.

Univariate and multivariate logistic regression models were used to estimate the association between the predictor variables (self-efficacy, positive mental health, and general health) and the outcomes (anxiety and depression), presented as odds ratios (OR) with a 95% confidence interval. Although logistic regression is traditionally recommended for rare outcomes, its application in cross-sectional studies has been accepted when the objective is to identify factors associated with dichotomous outcomes, even if prevalent. This model was chosen for its robustness and widespread use in studies with similar designs.

For continuous scores of self-efficacy, positive mental health, and general health, increments of 5% and 10% were calculated in relation to the lowest values on the scales, using the following formula: Percentage increment = [(Scale value / Minimum scale value) - 1)] × 100. Based on this percentage, variables representing 5% and 10% increments were created, which replaced the absolute values of the scales. This procedure allowed us to analyze how 5% and 10% increases in scale values, taking the lowest possible value as a reference, were associated with mental health outcomes.

For the multivariate models, the stepwise backward procedure was used, with initial inclusion of variables with p < 0.20 in the univariate analyses and permanence in the final model based on statistical significance and theoretical plausibility. This approach is recommended to avoid early exclusion of variables with potential explanatory relevance^(18)^.

The final models were adjusted as follows:

- Model for anxiety: adjusted for skin color, physical exercise, perception of stress, academic overload, and housing situation;
- Model for depression: adjusted for family relationship, satisfaction with the course, skin color, physical exercise, perception of stress, alcohol consumption, and weekly leisure activities.

### Ethical Aspects

This study is part of the matrix project “Positive mental health of higher education students in health,” cleared by the Research Ethics Committee of the São José do Rio Preto School of Medicine under Opinion No. 4,095,190 and Certificate of Ethical Presentation and Appraisal No. 31420620.8.0000. 5415. All procedures followed the guidelines of Resolutions No. 466/2012 and No. 510/2016 of the National Health Council. Data collection was conducted online, with acceptance recorded through an electronic Free and Informed Consent Form, previously presented in an explanatory video.

## RESULTS

A total of 138 nursing students participated in the study, predominantly female (94.2%), with a mean age of 21.6 years (SD = 4.7) and self-declared white (84.1%). Most did not have a partner (65.2%) and lived with their family (68.1%), while 23.2% lived with friends and 8.7% lived alone. Regarding their perception of financial resources, 87% considered that they had sufficient resources for their needs.

Regarding their academic experience, 90.6% of students reported feeling fulfilled with the course, although 89.1% felt overwhelmed by undergraduate activities.

Most did not have paid work (80.4%) and reported frequent feelings of stress (86.2%). Family relationships were classified as harmonious by 83.3% of participants. Only 42% reported practicing physical activity regularly, and 68.8% reported having weekly leisure activities.

Frequent alcohol consumption was reported by 14.5% of students, while smoking and illicit drug use were less prevalent, at 5.1% and 3.6%, respectively. Psychological or psychiatric counseling was mentioned by 31.9% of students.

Regarding mental health, 26% of participants reported having intentionally injured themselves at some point in their lives, with 10.1% during undergraduate studies and 15.9% prior to that. Suicidal ideation was reported by 39.9% of students, with 21% during undergraduate studies and 18.8% prior to entering the course.

The mean anxiety and depression scores were 10.72 (SD = 4.3) and 6.96 (SD = 3.93), respectively. According to the criteria adopted, 68.8% of students had symptoms of anxiety, and 37.0% had symptoms of depression.

The bivariate analysis showed significant associations between anxiety symptoms and sociodemographic and academic variables (Table 1). Students who lived alone had a higher prevalence of anxiety (p = 0.020), which may indicate greater emotional vulnerability associated with social isolation. Academic overload (p < 0.001), frequent stress (p < 0.001), and lack of weekly leisure activities (p = 0.001) also stood out as factors strongly associated with the presence of anxiety. In addition, conflictual family relationships (p = 0.040), lack of psychological or psychiatric follow-up (p = 0.008), history of self-harm (p = 0.001), and suicidal ideation (p < 0.001) before and during graduation were significantly associated with the outcome.

**Table 1:** Distribution of characteristics of students with anxiety and depression, according to sociodemographic and academic variables – São José do Rio Preto, SP, Brazil, 2020. (n = 138).

Variables	Anxiety n (%)	p-value	Depression n (%)	p-value
**Race**				
White	83 (87.4)	0.114	47 (92.2)	**0.047**
Non white	12 (12.6)		4 (7.8)	
**Living status**				
Living alone	12 (12.6)	**0.020**	7 (13.7)	0.134
Living with family	65 (68.4)		30 (5.8)	
Living with friends	18 (19.0)		14 (27.5)	
**Feel happy and fulfilled with undergraduate degree**				
No	10 (10.5)	0.509	9 (17.7)	**0.011**
Yes	85 (89.5)		42 (82.3)	
**Feeling overloaded by undergraduate activities**				
No	3 (3.2)	**<0.001**	2 (3.9)	**0.045**
Yes	92 (96.8)		49 (96.1)	
**Performs paid work**				
No	74 (77.9)	0.264	36 (70.6)	**0.026**
Yes	21 (22.1)		15 (29.4)	
**Feels frequently stressed**				
No	4 (4.2)	**<0.001**	1 (2.0)	**0.002**
Yes	91 (95.8)		50 (98.0)	
**Family relationships**				
Harmonious	75 (78.9)	**0.040**	37 (72.6)	**0.009**
Conflicting	20 (21.1)		14 (27.5)	
**Engages in leisure activities weekly**				
No	38 (40.0)	**0.001**	27 (52.9)	**<0.001**
Yes	57 (60.0)		24 (47.1)	
**Psychological or psychiatric counseling**				
No	58 (61.1)	**0.008**	29 (56.9)	**0.030**
Yes	37 (38.9)		22 (43.1)	
**Have you ever hurt yourself on purpose to relieve suffering?**				
No	62 (65.3)	**0.001**	34 (66.7)	0.138
Yes. during my undergraduate studies	14 (14.7)		5 (9.8)	
Yes. before my undergraduate studies	19 (20.0)		12 (23.5)	
**Had suicidal thoughts**				
No	44 (46.3)	**<0.001**	23 (45.1)	**0.017**
Yes. during my undergraduate studies	28 (29.5)		16 (31.4)	
Yes. before my undergraduate studies	23 (24.2)		12 (23.5)	

Regarding depression, the findings also revealed a set of factors associated with its higher prevalence. Of note is the association with white skin color (p = 0.047), lack of fulfillment with the course (p = 0.011), paid work concurrent with graduation (p = 0.026), academic overload (p = 0.045), stress (p = 0.002), conflictual family relationships (p = 0.009), lack of leisure time (p < 0.001), lack of psychological support (p = 0.030), and suicidal ideation (p = 0.017). Although self-harm was not statistically significant in relation to depression, the frequency of this behavior among students with depressive symptoms was significant.

### Correlation Matrix

As shown in Table 2, the results of the correlation matrix revealed significant associations between the scores of the different scales evaluated. The anxiety score showed a strong positive correlation with the depression score (r = 0.703; p < 0.001) and with the General Health Questionnaire 12 (QSG-12) score (r = 0.743; p < 0.001), indicating that higher levels of anxiety are associated with higher levels of depression and poorer general health. On the other hand, the anxiety score showed a significant negative correlation with the Perceived General Self-Efficacy Scale score (r = –0.520; p < 0.001) and with the total Positive Mental Health Questionnaire (QSMP) score (r = –0.216; p = 0.011), suggesting that higher anxiety is associated with lower self-efficacy and poorer positive mental health.

**Table 2:** Correlation matrix between the variables of anxiety, depression, general health, general self-efficacy, and positive mental health in nursing students – São José do Rio Preto, SP, Brazil, 2020.

	Anxiety score	Depression score	SMP total	QSG12 score	Self-efficacy score
Anxiety score	–	r: +0.703 p < 0.001	r: –0.216 p = 0.011	r: +0.743 p < 0.001	r: –0.520 p < 0.001
Depression score	r: +0.703 p < 0.001	–	r: –0.185 p = 0.030	r: +0.723 p < 0.001	r: –0.630 p < 0.001
SMP total	r: –0.216 p = 0.011	r: –0.185 p = 0.030	–	r: –0.172 p = 0.440	r: +0.166 p = 0.520
QSG12 score	r: +0.743 p < 0.001	r: +0.723 p < 0.001	r: –0.172 p = 0.440	–	r: –0.619 p < 0.001
Self-efficacy score	r: –0.520 p < 0.001	r: –0.630 p < 0.001	r: +0.166 p = 0.520	r: –0.619 p < 0.001	–

Spearman correlation test.

The depression score, also showed a significant negative ­correlation with the total PHMS score (r = –0.185; p = 0.030) and with the Perceived General Self-Efficacy Scale score (r = –0.630; p < 0.001), and a positive correlation with the ­QSG-12 score (r = 0.723; p < 0.001). This indicates that higher depression is associated with poorer positive mental health, lower self-efficacy, and poorer overall health.

In addition, the total QSMP score showed a negative, albeit insignificant, correlation with the QSG-12 score (r = –0.172; p = 0.440) and a non-significant positive correlation with the Perceived General Self-Efficacy Scale score (r = 0.166; p = 0.520). Finally, the QSG-12 score showed a significant negative correlation with the Perceived General Self-Efficacy Scale score (r = –0.619; p < 0.001), indicating that poorer general health is associated with lower self-efficacy.

### Logistic Regression

The results of the univariate logistic regressions, presented in Table 3, indicated that a 5% increase in the total QSMP score was associated with a 17.0% reduction in the probability of anxiety (OR = 0.83; 95% CI: 0.73–0.93) and a 21.0% reduction in the probability of depression (OR = 0.79; 95% CI: 0.70–0.89). For a 10.0% increase, these reductions were even greater, with a 32.0% lower probability of anxiety (OR = 0.68; 95% CI: 0.54–0.87) and a 38.0% lower probability of depression (OR = 0.62; 95% CI: 0.49–0.80).

**Table 3:** Univariate and multivariate logistic regression models for anxiety and depression in relation to general health, general self-efficacy, and positive mental health variables in nursing students – São José do Rio Preto, SP, Brazil, 2020.

	Anxiety		Depression	
	Univariate	Multivariate^1^	Univariate	Multivariate^2^
**SMP total**				
Increase 5%	0.86 (0.78–0.94)	0.83 (0.73–0.93)	0.82 (0.75–0.91)	0.79 (0.70–0.89)
Increase 10%	0.73 (0.61–0.89)	0.68 (0.54–0.87)	0.68 (0.56–0.82)	0.62 (0.49–0.80)
**QSG12 score**				
Increase 5%	1.27 (1.17–1.38)	1.29 (1.18–1.42)	1.19 (1.12–1.27)	1.18 (1.11–1.26)
Increase 10%	1.62 (1.38–1.90)	1.67 (1.39–2.01)	1.42 (1.26–1.61)	1.39 (1.22–1.59)
**Self-efficacy score**				
Increase 5%	0.92 (0.88–0.95)	0.89 (0.84–0.95)	0.89 (0.85–0.93)	0.89 (0.85–0.94)
Increase 10%	0.83 (0.78–0.90)	0.80 (0.71–0.90)	0.79 (0.73–0.86)	0.79 (0.72–0.88)

^1^ Model for anxiety adjusted for skin color, physical exercise, perception of stress, academic overload, and housing situation;

^2^ Model for depression adjusted for family relationships, satisfaction with the course, skin color, physical exercise, perception of stress, alcohol consumption, and weekly leisure activities.

In the case of the QSG-12, a 5.0% increase in the score was associated with a 29.0% increase in the probability of anxiety (OR = 1.29; 95% CI: 1.18–1.42) and an 18.0% increase in the probability of depression (OR = 1.18; 95% CI: 1.11–1.26). For a 10.0% increase, these increases were 67.0% (OR = 1.67; 95% CI: 1.39–2.01) for anxiety and 39.0% (OR = 1.39; 95% CI: 1.22–1.59) for depression.

Self-efficacy showed a protective relationship with anxiety and depression. A 5.0% increase in self-efficacy was associated with an 11.0% reduction in the probability of anxiety (OR = 0.89; 95% CI: 0.84–0.95) and an 11.0% reduction in the probability of depression (OR = 0.89; 95% CI: 0.85–0.94). For a 10.0% increase, these reductions were 20.0% for anxiety (OR = 0.80; 95% CI: 0.71–0.90) and 21.0% for depression (OR = 0.79; 95% CI: 0.72–0.88).

These results indicate that better positive mental health and greater self-efficacy are associated with lower levels of anxiety and depression, while poorer general health is associated with higher levels of anxiety and depression.

## DISCUSSION

This study revealed a high prevalence of anxiety and depression symptoms among nursing students, associated with psychosocial factors such as academic overload, stress, lack of leisure time, and conflictual family relationships. The strong correlation between anxiety and depression highlights the overlap between these disorders and their negative impact on overall health.

The findings suggest that symptoms of anxiety and depression among students are related to multiple interrelated factors, including living conditions, social support, previous mental health, and academic dynamics. The magnitude of the associations found highlights the need for institutional actions aimed at promoting mental health, with a focus on prevention and care, especially in more vulnerable groups.

The reduced perception of self-efficacy among students with anxiety and depressive symptoms highlights how such conditions undermine confidence in their own abilities, impairing academic performance and well-being^(19)^. Thus, the need for interventions aimed at promoting self-efficacy and managing academic stress is reinforced.

Although most participants were financially stable and lived with their families, traditionally protective factors, the high prevalence of psychological distress indicates that these elements were not sufficient to mitigate the negative effects of the pandemic context. Furthermore, females showed greater vulnerability, as evidenced in previous studies^(20,21)^, which points to the need for policies sensitive to gender specificities.

Housing conditions were also relevant: living alone or with friends was associated with a higher prevalence of depressive symptoms, indicating loneliness as a risk factor^(22)^. The presence of self-harm (26%) and suicidal ideation (39.9%) among students is alarming, corroborating findings from other studies on the worsening of psychological distress during the pandemic^(7,23)^.

The negative correlation between self-efficacy and symptoms of anxiety and depression reinforces its protective role. Students with higher levels of self-efficacy tend to experience less emotional distress and demonstrate greater academic engagement in contexts where positive mental health is a determining factor^(19,24)^.

The statistically significant associations between positive mental health, self-efficacy, and academic engagement^(24)^ demonstrate that strengthening positive aspects is essential not only for individual well-being but also for academic success. However, the fact that 68.1% of students do not receive psychological counseling reveals the absence of adequate institutional support, intensifying psychological vulnerability.

Simultaneous work and study can have ambivalent effects: while some students report that the professional environment favors socialization and improves mental health^(25)^, for others it can intensify stress, especially when there is a lack of adaptive strategies.

When poorly managed, academic stress can trigger substance use as a form of relief, further damaging mental health^(26)^. Preventive measures, such as the implementation of emotional management techniques, for example, diaphragmatic breathing^(27)^, can be effective and low-cost.

In addition, studies indicate that structured interventions to promote self-efficacy, based on psychoeducation and positive mental health strategies, can significantly reduce levels of stress, anxiety, and depression in university students^(8,9)^. These programs, usually conducted in weekly sessions, demonstrate a positive impact even in short-term interventions^(9)^.

The data obtained corroborate the need for institutional programs that promote positive mental health, strengthening self-efficacy and resilience^(28)^. Strategies such as reflective writing, emotional support, and communication skills development are pointed out as effective in coping with academic stressors^(29)^.

Recent studies show that factors such as course, gender, and occupation influence mental health, with nursing students being particularly vulnerable^(26)^. This vulnerability requires specific institutional actions, integrating mental health care into the teaching-learning process^(30)^.

This study has some important limitations. The sample consisted of students from a single public state institution, with non-probabilistic convenience sampling, which may compromise representativeness and limit the generalization of findings to other regional or institutional realities. The cross-sectional nature of the research also prevents the establishment of causal relationships between the variables studied. In addition, online data collection, although necessary due to social distancing, may have generated selection bias, excluding students with less access to the internet or less engaged in academic activities.

Despite these limitations, this study offers relevant contributions to existing knowledge. By integrating variables that have been little explored together, such as positive mental health, self-efficacy, and psychological distress, the findings broaden the understanding of protective and risk factors among nursing students. The evidence obtained can support the development of institutional programs focused on promoting well-being, preventing emotional disorders, and strengthening social-emotional skills in health education.

## CONCLUSION

The study showed a high prevalence of anxiety and depression among nursing students, associated with multiple psychosocial and academic factors, such as overload, frequent stress, lack of leisure time, and limited psychological support. The perception of self-efficacy and positive mental health demonstrated a relevant protective role, correlating negatively with symptoms of psychological distress and positively with well-being and academic engagement.

The results presented offer useful evidence to guide pedagogical practices and institutional policies focused on mental health in higher education. They also point to viable paths for psychoeducational interventions, prevention actions, and strengthening emotional support in the academic environment, especially when articulated with structured strategies that promote healthier academic environments, focusing on reducing stressors, strengthening social-emotional skills, promoting positive mental health, and expanding access to psychological support. Incorporating these actions into the pedagogical design of nursing courses can benefit not only students’ health but also their ethical, critical, and resilient training in the face of the challenges of the profession.

We recommend the development of new studies with larger, multicenter samples and longitudinal designs, capable of deepening the understanding of the causal relationships between mental health, self-efficacy, and academic performance, especially in highly vulnerable contexts such as the post-pandemic period.

## Data Availability

The data supporting the results of this study are available upon request to the corresponding author, due to ethical approval requirements.

## References

[1] Gomes IM, Silva RB. (2021). Universitários ingressantes: expectativas e dificuldades na adaptação à vida acadêmica. Pró-Discente.

[2] Reverté-Villarroya S, Ortega L, Sánchez-Guarnido AJ. (2021). Stress, anxiety, and depression in nursing students: a systematic review. J Nurs Manag.

[3] Ribeiro IJS, Pereira R, Freire IV, Oliveira BG, Casotti CA, Boery EN. (2020). Stress and quality of life among university students: a systematic literature review. Health Prof Educ.

[4] Aquino JP, Cardoso AS, Pinho LB. (2019). A saúde mental de estudantes universitários: reflexões para o cuidado em saúde. Rev Bras Enferm.

[5] Lourenço RG, Andrade MS, Silva RA. (2021). Educação em tempos de pandemia: desafios e perspectivas no ensino superior. Rev Bras Educ.

[6] Vital LP. (2024). Saúde mental dos estudantes de enfermagem no pós-pandemia: um olhar necessário. Rev Bras Enferm.

[7] Oliveira EN, Vasconcelos MIO, Almeida PC, Pereira PJA, Linhares MSC, Ximenes No FRG (2022). Covid-19: Repercussions on the mental health of higher education students. Saúde Debate.

[8] Alves DM, Pompeo DA, Sacardo Y, Eid LP, Lourenção LG, André JC. (2024). Influence of self-efficiency beliefs on the health and well-being of university students in COVID-19. Rev Gaúcha Enferm.

[9] Gueroni LPG, Pompeo DA, Eid LP, Ferreira MA, Sequeira CAC, Lourenção LG. (2024). Interventions for strengthening general self-efficacy beliefs in college students: an integrative review. Rev Bras Enferm.

[10] Santiago J, Bernaras E, Jaureguizar J (2020). Salud mental positiva: del concepto al constructo. Evolución histórica y revisión de teorías. Rev Port Enferm Saúde Ment.

[11] Norabuena-Figueroa RP, Rodríguez-Orellana HM, Norabuena-Figueroa ED, Deroncele-Acosta A. (2025). Organizational climate as a key to positive mental health and academic engagement in university students: a structural equation modeling approach. Eur J Investig Health Psychol Educ.

[12] Marcolino JA, Mathias LA, Piccinini Fo L, Guaratini AA, Suzuki FM, Alli LA. (2007). Hospital Anxiety and Depression Scale: a study on the validation of the criteria and reliability on preoperative patients. Rev Bras Anestesiol.

[13] Balsan LAG, Carneiro LL, Bastos AVB, Costa VMF. (2020). Adaptação e validação da nova escala geral de autoeficácia. Aval Psicol.

[14] Goldberg DP, Williams PA. (1988). A user’s guide to the General Health Questionnaire.

[15] Sequeira C, Carvalho JC, Sampaio F, Sá L, Lluch-Canut MT, Roldán-Merino J. (2014). Avaliação das propriedades psicométricas do Questionário de Saúde Mental Positiva em estudantes portugueses do ensino superior. Rev Port Enferm Saúde Ment.

[16] Pasquali L, Gouveia VV, Andriola WB, Miranda FJ, Ramos ALM. (1994). Questionário de saúde geral de Goldberg (QSG): adaptação brasileira. Psicol Teor Pesqui.

[17] Gouveia VV, Pasquali L, Andriola WB, Miranda FJ, Ramos ALM. (2010). Factorial validity and reliability of the General Health Questionnaire (GHQ-12) in the Brazilian physician population. Cad Saude Publica.

[18] Hosmer DW, Lemeshow S (2000). Applied logistic regression.

[19] Melo HE, Severian PFG, Eid LP, Souza MR, Sequeira CAC, Souza MGG (2021). Impact of anxiety and depression symptoms on perceived self-efficacy in nursing students. Acta Paul Enferm.

[20] Ardisson GMC, Andrade RO, Andrião AV, Mafra AC, Fonseca MCKL, Amâncio MG (2021). Saúde mental e qualidade de vida dos estudantes de faculdades de medicina brasileiras: uma revisão integrativa. REAS.

[21] Teodoro MLM, Gomes JS, Batista JBV, Santos CG. (2021). Saúde mental em estudantes universitários durante a pandemia de covid-19. Rev Família Ciclos Vida Saúde Contexto Soc.

[22] Maia HAAS, Assunção ACS, Silva CS, Santos JLP, Menezes CJJ, de Bessa J. (2020). Prevalência de sintomas depressivos em estudantes de medicina com currículo de aprendizagem baseada em problemas. Rev Bras Educ Med.

[23] Martínez-Esquivel D, Quesada-Carballo P, Quesada-Rodríguez Y, Muñoz-Rojas D, Solano-López AL. (2022). Relação entre a depressão e o apoio social percebido nos estudantes de enfermagem no contexto de comportamentos suicidas. Cogitare Enferm.

[24] Amaral EL, Frick LT. (2022). Correlation between academic engagement and positive mental health in university students. Rev Int Educ Super.

[25] Nazari AM, Borhani F, Abbaszadeh A, Kangarbani MAK. (2025). Self-compassion, academic stress, and academic self-efficacy among undergraduate nursing students: a cross-sectional, multi-center study. BMC Med Educ.

[26] Graner KM, Cerqueira ATAR. (2019). Revisão integrativa: sofrimento psíquico em estudantes universitários e fatores associados. Ciênc Saúde Coletiva.

[27] Ribeiro A, Vicente C, Santos K, Carvalho M, Mendes S, Seabra P. (2023). O relaxamento como técnica de redução da ansiedade da pessoa com doença mental: revisão integrativa da literatura. Rev Port Enferm Saúde Ment.

[28] Alves S, Ribeiro I, Sequeira C (2021). Criação e validação de um programa promotor de saúde mental positiva em adolescentes. Rev ROL Enferm.

[29] Rodrigues TCMM, Barbosa GC, Tonete VLP. (2022). Coping with the psychological distress of students in the university context: an integrative review. Rev Fam Ciclos Vida Saúde Contexto Soc.

[30] Martins RCC, Branco RPC. (2021). The impacts of mental health on university students in the Nursing course: literature review. Res Soc Dev.

