# Supplementary material for: General health, anxiety, and depression: relationship with perceived self-efficacy and positive mental health in nursing students
Source: Rev Esc Enferm USP. 2025 Nov 17;59:e20250260. doi: 10.1590/1980-220X-REEUSP-2025-0260en (PMC12625990; doi:10.1590/1980-220X-REEUSP-2025-0260en)
Supplement: Translation

# Translation: Saúde geral, ansiedade e depressão: relação com autoeficácia percebida e saúde mental positiva em estudantes de enfermagem*

**DOI:** 10.1590/1980-220X-REEUSP-2025-0260pt

**Authors:** Eliel de Oliveira Bandeira, Daniele Alcalá Pompeo, Natália Sperli Geraldes Marin dos Santos Sasaki, Camila Garcel Pancote, Luiz Antônio Alves de Menezes-Júnior, Danilo de Miranda Alves, Letícia Palota Eid, Luciano Garcia Lourenção

**Affiliations:** 1 Universidade Federal do Rio Grande, Escola de Enfermagem, Rio Grande, RS, Brasil.; 2 Faculdade de Medicina de São José do Rio Preto, São José do Rio Preto, SP, Brasil.; 3 União das Faculdades dos Grandes Lagos, São José do Rio Preto, SP, Brasil.; 4 Universidade Federal de Ouro Preto, Escola de Nutrição, Ouro Preto, MG, Brasil.; 5 Universidade Federal de Jataí, Instituto de Ciências da Saúde, Jataí, GO, Brasil.

**Keywords:** Estudantes de Enfermagem, Saúde Mental, Autoeficácia, Ansiedade, Depressão

## Abstract

**Objetivo:**

Analisar as condições de saúde geral, ansiedade e depressão de estudantes de enfermagem e analisar a sua relação com a percepção de autoeficácia geral e saúde mental positiva, durante o enfrentamento da covid-19.

**Método:**

Estudo transversal, descritivo e correlacional, com 138 estudantes de enfermagem de uma instituição pública. Aplicaram-se instrumentos para variáveis sociodemográficas, Escala Hospitalar de Ansiedade e Depressão, Escala de Autoeficácia Geral, Questionário de Saúde Geral e de Saúde Mental Positiva. As análises incluíram estatística descritiva, correlações e regressão logística univariada e multivariada.

**Resultados:**

Um aumento de 5% no escore de Saúde Mental Positiva associou-se à redução de 20,4% na probabilidade de ansiedade (OR = 0,83; IC 95%: 0,73–0,93) e de 26,6% na de depressão (OR = 0,79; IC 95%: 0,70–0,89). Incrementos semelhantes no Questionário de Saúde Geral foram associados a maiores probabilidades de ansiedade (29%) e depressão (18%). Um aumento de 5% na percepção de autoeficácia geral reduziu em 12,4% a probabilidade de ambos os desfechos (ansiedade e depressão).

**Conclusão:**

Saúde mental positiva e autoeficácia foram fatores protetores, reforçando a importância de ações institucionais para promover o bem-estar acadêmico.

## INTRODUÇÃO

A transição para a vida universitária representa uma etapa crítica no desenvolvimento dos jovens adultos, marcada por mudanças significativas no modelo de ensino-aprendizagem, nas interações sociais e na estrutura emocional^(1)^. Para estudantes da área da saúde, como os de enfermagem, essa adaptação envolve ainda a vivência de situações emocionalmente exigentes e de alta responsabilidade, que podem comprometer o bem-estar psicológico e aumentar a vulnerabilidade para o desenvolvimento de transtornos mentais, como ansiedade e depressão^(2)^.

Diversos estudos apontam que estudantes de enfermagem apresentam níveis elevados de sofrimento psíquico em comparação com colegas de outros cursos, em decorrência da carga horária extensa, da intensidade das atividades práticas e do contato precoce com situações de dor, sofrimento e morte^(3)^. Essas condições tornam esse grupo especialmente suscetível ao adoecimento mental, com implicações negativas sobre o desempenho acadêmico, o risco de evasão e a qualidade da formação profissional^(2,4)^.

A pandemia de covid-19 agravou esse cenário, ao impor medidas de isolamento social e transformações abruptas no processo de ensino-aprendizagem, com a migração para o ensino remoto e a suspensão de atividades práticas essenciais para a formação em enfermagem^(5)^. Investigações relataram o aumento expressivo de sintomas de ansiedade, estresse e depressão entre estudantes universitários, especialmente os da área da saúde, que passaram a ser reconhecidos como um grupo prioritário para ações de promoção e cuidado em saúde mental^(6,7)^.

Nesse contexto, a autoeficácia, definida como a crença na capacidade de organizar e executar ações para alcançar determinados objetivos, tem se destacado como um fator de proteção para a saúde mental de estudantes universitários^(8)^. Indivíduos com altos níveis de autoeficácia tendem a encarar desafios acadêmicos como oportunidades de crescimento, apresentando maior resiliência emocional, menor reatividade ao estresse e menor propensão à ansiedade e à depressão^(9)^.

A Saúde Mental Positiva (SMP), por sua vez, representa uma abordagem centrada no bem-estar psicológico que ultrapassa a mera ausência de doença mental. Envolve aspectos como satisfação pessoal, autocontrole, habilidades de relacionamento, autonomia e realização pessoal^(10)^. A literatura tem enfatizado a importância de sua promoção desde os primeiros momentos da formação acadêmica, como estratégia para fortalecer competências socioemocionais e prevenir o sofrimento psíquico^(11)^.

Apesar dos avanços na produção científica sobre saúde mental de universitários, ainda são escassos os estudos que analisam, de forma integrada, os vínculos entre saúde geral, sintomas emocionais (ansiedade e depressão), autoeficácia percebida e saúde mental positiva, sobretudo entre estudantes de enfermagem no Brasil. Essa lacuna se evidencia ainda mais diante do impacto desproporcional da pandemia sobre esse grupo e da necessidade de gerar evidências que fundamentem ações institucionais de acolhimento, prevenção e promoção do bem-estar universitário^(6)^.

Este estudo busca ampliar a literatura ao examinar, de forma integrada, variáveis tradicionalmente analisadas de modo isolado, como saúde mental positiva, autoeficácia percebida, ansiedade, depressão e saúde geral, contribuindo para uma compreensão mais abrangente da saúde mental de estudantes de Enfermagem. Parte-se da hipótese de que níveis mais elevados de saúde mental positiva e de autoeficácia estão associados a menores níveis de ansiedade e depressão, enquanto piores condições de saúde geral estão relacionadas a maiores riscos de sofrimento emocional.

Ante o exposto, o estudo objetivou analisar as condições de saúde geral, ansiedade e depressão de estudantes de enfermagem e analisar a sua relação com a percepção de autoeficácia geral e saúde mental positiva, durante o enfrentamento da covid-19.

## MÉTODO

### Desenho Do Estudo

Trata-se de um estudo transversal, descritivo e correlacional, conduzido com estudantes de enfermagem de uma instituição de ensino superior pública estadual do interior do estado de São Paulo, Brasil. O manuscrito foi estruturado conforme as diretrizes da ferramenta STROBE (Strengthening the Reporting of Observational Studies in Epidemiology).

### Local do Estudo

O estudo foi realizado na Faculdade de Medicina de São José do Rio Preto (Famerp), que oferta os cursos de graduação em Medicina, Enfermagem e Psicologia, além de cursos de Pós-graduação Lato Sensu e Stricto Sensu. A escolha da instituição se deu em razão de sua relevância na formação de profissionais de saúde e do aumento da demanda por serviços de apoio psicológico entre seus estudantes, observada especialmente durante a pandemia de covid-19.

### População, Critérios de Seleção E Amostra

A população do estudo incluiu 190 estudantes matriculados no curso de Enfermagem, no ano de 2020. A identificação dos participantes ocorreu por meio de listagem fornecida pela instituição de ensino. Os critérios de inclusão estabelecidos foram: estar regularmente matriculado no curso de graduação em enfermagem da instituição; ter idade ≥ 18 anos e estar presente nas datas de coletas de dados.

Para o cálculo amostral, considerou-se um nível de confiança de 95%, margem de erro de 5% e proporção esperada de 50%, resultando em uma amostra mínima estimada de 128 estudantes.

Adotou-se uma amostragem não probabilística, por conveniência, com inclusão de todos os estudantes que consentiram em participar e responderam integralmente aos instrumentos. Participaram do estudo 138 estudantes, representando 72,6% da população total, o que assegura uma proporção adequada para os objetivos do estudo.

### Coleta de Dados

Para a coleta de dados, foram utilizados cinco instrumentos autoaplicáveis: um questionário contendo variáveis pessoais, sociais, acadêmicas, hábitos de vida, além de questionamentos sobre presença de estresse, sobrecarga e percepção do impacto da pandemia em sua vida; a Escala Hospitalar de Ansiedade e Depressão (*Hospital Anxiety and Depression Scale* – HADS)^(12)^; a Escala de Autoeficácia Geral e Percebida^(13)^; o Questionário de Saúde Geral (QSG-12)^(14)^; e o Questionário de Saúde Mental Positiva (QSMP)^(15)^.

A HADS mensura sintomas sugestivos de ansiedade e depressão em ambientes não psiquiátricos, baseada na última semana do indivíduo. Cada item da HADS contempla quatro possibilidades de respostas (0 a 3), totalizando pontuação máxima de 21 pontos para cada subescala^(12)^. Estudantes com escore ≥ 9 em cada subescala foram considerados com ansiedade e/ou com depressão.

A Escala de Autoeficácia Geral e Percebida é composta por 10 itens, com respostas que variam de um a cinco, em que 1 significa discordo totalmente e 5 concordo totalmente. Cada item mensura as crenças que o indivíduo possui acerca de suas capacidades para alcançar um objetivo, lidar com uma situação ou desempenhar uma tarefa (Exemplo: Eu sempre consigo resolver os problemas difíceis se eu tentar bastante; é fácil para mim, agarrar-me aos meus objetivos e atingir as minhas metas). A pontuação se dá por meio da soma dos valores dos itens e maior pontuação indica maior percepção da autoeficácia, em um intervalo de 10 a 50^(13)^.

O QSG-12 corresponde a uma versão abreviada do Questionário de Saúde Geral, inicialmente elaborado com 60 itens. A versão com 12 itens é a menor e a mais empregada para mensurar o bem-estar psicológico, já que as evidências demonstraram que a redução do número de itens não comprometeu a confiabilidade das respostas. O QSG-12 é composto de 12 itens que avaliam o quanto a pessoa tem experimentado os sintomas descritos, com quatro possibilidades de respostas. Nos itens que negam a saúde mental, as alternativas de respostas podem ser: (1) absolutamente não, (2) não mais que de costume, (3) um pouco mais que de costume e (4) muito mais que de costume. Nos itens positivos, as respostas variam de: (1) mais que de costume, (2) o mesmo de sempre, (3) menos que de costume, (4) muito menos que de costume. Observa-se que os itens negativos foram invertidos e a menor pontuação indica melhor nível de bem-estar^(14,16)^. No Brasil, o QGS-12 tem demonstrado evidências de validade e precisão, além de consistência interna, avaliada de alfa de Cronbach acima de 0,80^(17)^.

O Questionário de Saúde Mental Positiva (QSMP) mede a saúde mental global positiva, por meio de 39 afirmações sobre forma de pensar, sentir e agir de cada indivíduo^(15)^. Essas afirmativas foram agrupadas em seis fatores: (F1) satisfação pessoal, (F2) atitude positiva, (F3) autocontrole, (F4) autonomia, (F5) realização pessoal e resolução de problemas e (F6) habilidades de relacionamento interpessoal. As respostas a cada item são apresentadas em uma escala Likert de 4 pontos: (1) sempre ou quase sempre, (2) na maioria das vezes, (3) algumas vezes, (4) quase nunca ou nunca. Alguns itens do instrumento estão descritos de forma positiva (n = 19) e outros de forma negativa (n = 20), na tentativa de se identificar possíveis falhas no preenchimento, como a automaticidade na marcação dos itens. As pontuações do QSMP variam de 39 (pontuação mínima) a 156 (pontuação máxima). Quanto maior a pontuação obtida, maior o nível de saúde mental positiva^(15)^. A versão portuguesa foi validada em uma amostra de 942 universitários, com bons resultados de consistência interna (alfa de Cronbach = 0,92) e uma estrutura fatorial semelhante ao estudo original^(15)^.

Os dados foram coletados no segundo semestre de 2020. O convite para participar do estudo foi encaminhado a todos os alunos do curso de enfermagem, para o e-mail fornecido no ato da matrícula e disponibilizado pela instituição, e por mensagem de *WhatsApp* para os grupos das turmas de graduação. O convite consistia em um vídeo informativo que apresentava o título da pesquisa, seus objetivos, os responsáveis pela condução e os instrumentos que deveriam ser respondidos, além de ressaltar que a participação era voluntária e que o estudo atendia as recomendações éticas para estudos com seres humanos.

Ao concordarem participar do estudo, os estudantes clicavam em um *link* disponibilizado no final do vídeo, que direcionava para o Termo de Consentimento Livre e Esclarecido (TCLE). Após a leitura, os estudantes tinham a opção de aceitar participar do estudo. Depois de confirmarem o aceite, os estudantes tinham acesso aos instrumentos de coleta dos dados (questionário sociodemográfico, HADS, Escala de Autoeficácia Geral e Percebida, QSG-12 e QSMP, todos disponibilizados por meio da plataforma *Google Forms*. O uso dessa ferramenta foi essencial para a coleta dos dados, devido à realização do estudo no período de isolamento social decorrente do quadro sanitário gerado pela pandemia de covid-19, em que os estudantes estavam afastados das atividades *in loco*.

### Análise e Tratamento dos Dados

A análise estatística foi realizada com o software Stata® 15.0, adotando-se nível de significância de 5%. Inicialmente, foram realizadas análises descritivas (frequências, médias e desvios-padrão) e testes bivariados (qui-quadrado de Pearson e t de Student) para comparação dos desfechos de ansiedade e depressão com variáveis sociodemográficas. A correlação entre os escores da HADS, QSG-12, Escala de Autoeficácia e QSMP foi avaliada pelo coeficiente de Spearman.

Modelos de regressão logística univariada e multivariada foram empregados para estimar a associação entre as variáveis preditoras (autoeficácia, saúde mental positiva e saúde geral) e os desfechos (ansiedade e depressão), apresentados em forma de odds ratios (OR) com intervalo de confiança de 95%. Apesar de a regressão logística ser tradicionalmente recomendada para desfechos raros, sua aplicação em estudos transversais tem sido aceita quando o objetivo é identificar fatores associados a desfechos dicotômicos, ainda que prevalentes. Optou-se por esse modelo pela sua robustez e ampla utilização em pesquisas com delineamento semelhante.

Para os escores contínuos de autoeficácia, saúde mental positiva e saúde geral, foram calculados incrementos de 5% e 10% em relação aos menores valores das escalas, a partir da seguinte fórmula: Percentual de incremento = [(Valor da escala / Valor mínimo da escala) - 1)] × 100. A partir desse percentual, criou-se variáveis que representavam incrementos de 5% e 10%, que substituíram os valores absolutos das escalas. Esse procedimento permitiu analisar como aumentos de 5% e 10% nos valores das escalas, tomando como referência o menor valor possível, se associavam aos desfechos de saúde mental.

Para os modelos multivariados, foi utilizado o procedimento *stepwise backward*, com inclusão inicial de variáveis com p < 0,20 nas análises univariadas e permanência no modelo final com base na significância estatística e na plausibilidade teórica. Essa abordagem é recomendada para evitar exclusão precoce de variáveis com potencial relevância explicativa^(18)^.

Os modelos finais foram ajustados da seguinte forma:

- Modelo para ansiedade: ajustado por cor de pele, prática de exercício físico, percepção de estresse, sobrecarga acadêmica e situação de moradia;
- Modelo para depressão: ajustado por relação familiar, realização com o curso, cor da pele, prática de exercício físico, percepção de estresse, uso de bebidas alcoólicas e prática de lazer semanal.

### Aspectos Éticos

Este estudo faz parte do projeto matricial “*Saúde mental positiva de estudantes do ensino superior em saúde*”, aprovado pelo Comitê de Ética em Pesquisa da Faculdade de Medicina de São José do Rio Preto sob Parecer nº 4.095.190 e Certificado de Apresentação e Apreciação Ética nº 31420620.8.0000.5415. Todos os procedimentos seguiram as diretrizes das Resoluções nº 466/2012 e nº 510/2016, do Conselho Nacional de Saúde. A coleta foi realizada de forma online, com aceite registrado por meio do Termo de Consentimento Livre e Esclarecido eletrônico, previamente apresentado em vídeo explicativo.

## RESULTADOS

Participaram do estudo 138 estudantes de Enfermagem, com predomínio do sexo feminino (94,2%), média de idade de 21,6 anos (DP = 4,7) e autodeclarados brancos (84,1%). A maioria não possuía companheiro (65,2%) e morava com a família (68,1%), enquanto 23,2% residiam com amigos e 8,7% moravam sozinhos. Quanto à percepção sobre recursos financeiros, 87% consideraram ter recursos suficientes para suas necessidades.

Em relação à vivência acadêmica, 90,6% dos estudantes relataram sentir-se realizados com o curso, embora 89,1% se sentissem sobrecarregados com as atividades da graduação. A maioria não exercia trabalho remunerado (80,4%) e referiu sensação frequente de estresse (86,2%). A relação familiar foi classificada como harmoniosa por 83,3% dos participantes.

Apenas 42% relataram praticar atividade física regularmente, e 68,8% afirmaram ter lazer semanal. O uso frequente de bebidas alcoólicas foi referido por 14,5% dos estudantes, enquanto o tabagismo e o uso de drogas ilícitas foram menos prevalentes, com 5,1% e 3,6%, respectivamente. Acompanhamento psicológico ou psiquiátrico foi mencionado por 31,9% dos estudantes.

Com relação à saúde mental, 26% dos participantes relataram ter se ferido propositalmente em algum momento da vida, sendo 10,1% durante a graduação e 15,9% anteriormente. A ideação suicida foi referida por 39,9% dos estudantes, sendo 21% durante a graduação e 18,8% antes do ingresso no curso.

A média dos escores de ansiedade e depressão foi de 10,72 (DP = 4,3) e 6,96 (DP = 3,93), respectivamente. De acordo com os critérios adotados, 68,8% dos estudantes apresentavam sintomas de ansiedade, e 37,0%, de depressão.

A análise bivariada evidenciou associações significativas entre os sintomas de ansiedade e variáveis sociodemográficas e acadêmicas (Tabela 1). Estudantes que moravam sozinhos apresentaram maior prevalência de ansiedade (p = 0,020), o que pode indicar maior vulnerabilidade emocional associada ao isolamento social. A sobrecarga acadêmica (p < 0,001), o estresse frequente (p < 0,001) e a ausência de lazer semanal (p = 0,001) também se destacaram como fatores fortemente associados à presença de ansiedade. Além disso, relações familiares conflituosas (p = 0,040), ausência de acompanhamento psicológico ou psiquiátrico (p = 0,008), histórico de automutilação (p = 0,001) e ideação suicida (p < 0,001) antes e durante a graduação estiveram significativamente associados ao desfecho.

**Tabela 1:** Distribuição das características dos estudantes com ansiedade e depressão, segundo variáveis sociodemográficas e acadêmicas – São José do Rio Preto, SP, Brasil, 2020. (n = 138).

Variáveis	Ansiedade n (%)	p-valor	Depressão n (%)	p-valor
**Cor da pele**				
Branco	83 (87,4)	0,114	47 (92,2)	**0,047**
Não branco	12 (12,6)		4 (7,8)	
**Situação de Moradia**				
Mora Sozinho	12 (12,6)	**0,020**	7 (13,7)	0,134
Mora com Família	65 (68,4)		30 (5,8)	
Mora com Amigos	18 (19,0)		14 (27,5)	
**Sente-se feliz e realizado com o curso de graduação**				
Não	10 (10,5)	0,509	9 (17,7)	**0,011**
Sim	85 (89,5)		42 (82,3)	
**Sente-se sobrecarregado com as atividades da graduação**				
Não	3 (3,2)	**<0,001**	2 (3,9)	**0,045**
Sim	92 (96,8)		49 (96,1)	
**Realiza trabalho remunerado**				
Não	74 (77,9)	0,264	36 (70,6)	**0,026**
Sim	21 (22,1)		15 (29,4)	
**Sente-se estressado com frequência**				
Não	4 (4,2)	**<0,001**	1 (2,0)	**0,002**
Sim	91 (95,8)		50 (98,0)	
**Relação com a família**				
Harmoniosa	75 (78,9)	**0,040**	37 (72,6)	**0,009**
Conflituosa	20 (21,1)		14 (27,5)	
**Pratica atividades de lazer semanalmente**				
Não	38 (40,0)	**0,001**	27 (52,9)	**<0,001**
Sim	57 (60,0)		24 (47,1)	
**Acompanhamento psicológico ou psiquiátrico**				
Não	58 (61,1)	**0,008**	29 (56,9)	**0,030**
Sim	37 (38,9)		22 (43,1)	
**Já se feriu propositalmente para aliviar um sofrimento**				
Não	62 (65,3)	**0,001**	34 (66,7)	0,138
Sim, durante a graduação	14 (14,7)		5 (9,8)	
Sim, antes da graduação	19 (20,0)		12 (23,5)	
**Teve ideação suicida**				
Não	44 (46,3)	**<0,001**	23 (45,1)	**0,017**
Sim, durante a graduação	28 (29,5)		16 (31,4)	
Sim, antes da graduação	23 (24,2)		12 (23,5)	

Em relação à depressão, os achados também revelaram um conjunto de fatores associados à sua maior prevalência. Destaca-se a associação com a cor da pele branca (p = 0,047), ausência de realização com o curso (p = 0,011), trabalho remunerado concomitante à graduação (p = 0,026), sobrecarga acadêmica (p = 0,045), estresse (p = 0,002), relação familiar conflituosa (p = 0,009), ausência de lazer (p < 0,001), ausência de acompanhamento psicológico (p = 0,030) e ideação suicida (p = 0,017). Embora a automutilação não tenha apresentado significância estatística em relação à depressão, a frequência do comportamento entre os estudantes com sintomas depressivos foi expressiva.

### Matriz de Correlação

Conforme demonstra a Tabela 2, os resultados da matriz de correlação revelaram associações significativas entre os escores das diferentes escalas avaliadas. O escore de ansiedade apresentou uma forte correlação positiva com o escore de depressão (r = 0,703; p < 0,001) e com o escore do Questionário de Saúde Geral 12 (QSG-12) (r = 0,743; p < 0,001), indicando que níveis mais altos de ansiedade estão associados a níveis mais altos de depressão e pior saúde geral. Por outro lado, o escore de ansiedade mostrou uma correlação negativa significativa com o escore da Escala de Autoeficácia Geral Percebida (r = –0,520; p < 0,001) e com o escore do Questionário de Saúde Mental Positiva (QSMP) total (r = –0,216; p = 0,011), sugerindo que maior ansiedade está associada a menor autoeficácia e pior saúde mental positiva.

**Tabela 2:** Matriz de correlação entre as variáveis de ansiedade, depressão, saúde geral, autoeficácia geral e saúde mental positiva nos estudantes de enfermagem – São José do Rio Preto, SP, Brasil, 2020.

	Escore Ansiedade	Escore Depressão	SMP total	Escore QSG12	Escore Autoeficácia
Escore Ansiedade	–	r: +0,703 p < 0,001	r: –0,216 p = 0,011	r: +0,743 p < 0,001	r: –0,520 p < 0,001
Escore Depressão	r: +0,703 p < 0,001	–	r: –0,185 p = 0,030	r: +0,723 p < 0,001	r: –0,630 p < 0,001
SMP total	r: –0,216 p = 0,011	r: –0,185 p = 0,030	–	r: –0,172 p = 0,440	r: +0,166 p = 0,520
Escore QSG12	r: +0,743 p < 0,001	r: +0,723 p < 0,001	r: –0,172 p = 0,440	–	r: –0,619 p < 0,001
Escore Autoeficácia	r: –0,520 p < 0,001	r: –0,630 p < 0,001	r: +0,166 p = 0,520	r: –0,619 p < 0,001	–

Teste de Correlação de Spearman.

O escore de depressão também apresentou correlação negativa significativa com o escore do QSMP total (r = –0,185; p = 0,030) e com o escore da Escala de Autoeficácia Geral Percebida (r = –0,630; p < 0,001), e uma correlação positiva com o escore do QSG–12 (r = 0,723; p < 0,001). Isso indica que maior depressão está associada a pior saúde mental positiva, menor autoeficácia e pior saúde geral.

Além disso, o escore do QSMP total apresentou uma correlação negativa, embora não significativa, com o escore do QSG-12 (r = –0,172; p = 0,440) e uma correlação positiva não significativa com o escore da Escala de Autoeficácia Geral Percebida (r = 0,166; p = 0,520). Por fim, o escore do QSG-12 mostrou uma correlação negativa significativa com o escore da Escala de Autoeficácia Geral Percebida (r = –0,619; p < 0,001), indicando que pior saúde geral está associada a menor autoeficácia.

### Regressão Logística

Os resultados das regressões logísticas univariadas, apresentados na Tabela 3, indicaram que o incremento de 5% no escore do QSMP total estava associado a uma redução de 17,0% na probabilidade de ansiedade (OR = 0,83; IC 95%: 0,73–0,93) e uma redução de 21,0% na probabilidade de depressão (OR = 0,79; IC 95%: 0,70–0,89). Para um incremento de 10,0%, essas reduções foram ainda maiores, com 32,0% menor probabilidade de ansiedade (OR = 0,68; IC 95%: 0,54–0,87) e 38,0% menor probabilidade de depressão (OR = 0,62; IC 95%: 0,49–0,80).

**Tabela 3:** Modelos de regressão logística univariada e multivariada para ansiedade e depressão em relação às variáveis de saúde geral, autoeficácia geral e saúde mental positiva nos estudantes de enfermagem – São José do Rio Preto, SP, Brasil, 2020.

	Ansiedade		Depressão	
	Univariada	Multivariada^1^	Univariada	Multivariada^2^
**SMP total**				
Incremento 5%	0,86 (0,78–0,94)	0,83 (0,73–0,93)	0,82 (0,75–0,91)	0,79 (0,70–0,89)
Incremento 10%	0,73 (0,61–0,89)	0,68 (0,54–0,87)	0,68 (0,56–0,82)	0,62 (0,49–0,80)
**Escore QSG12**				
Incremento 5%	1,27 (1,17–1,38)	1,29 (1,18–1,42)	1,19 (1,12–1,27)	1,18 (1,11–1,26)
Incremento 10%	1,62 (1,38–1,90)	1,67 (1,39–2,01)	1,42 (1,26–1,61)	1,39 (1,22–1,59)
**Escore Autoeficácia**				
Incremento 5%	0,92 (0,88–0,95)	0,89 (0,84–0,95)	0,89 (0,85–0,93)	0,89 (0,85–0,94)
Incremento 10%	0,83 (0,78–0,90)	0,80 (0,71–0,90)	0,79 (0,73–0,86)	0,79 (0,72–0,88)

^1^ Modelo para ansiedade ajustado por cor de pele, prática de exercício físico, percepção de estresse, sobrecarga acadêmica e situação de moradia;

^2^ Modelo para depressão ajustado por relação familiar, realização com o curso, cor da pele, prática de exercício físico, percepção de estresse, uso de bebidas alcoólicas e prática de lazer semanal.

No caso do QSG-12, um incremento de 5,0% no escore estava associado a um aumento de 29,0% na probabilidade de ansiedade (OR = 1,29; IC 95%: 1,18–1,42) e um aumento de 18,0% na probabilidade de depressão (OR = 1,18; IC 95%: 1,11–1,26). Para um incremento de 10,0%, esses aumentos foram de 67,0% (OR = 1,67; IC 95%: 1,39–2,01) para ansiedade e 39,0% (OR = 1,39; IC 95%: 1,22–1,59) para depressão.

A autoeficácia mostrou uma relação protetora em relação à ansiedade e depressão. Um incremento de 5,0% na autoeficácia estava associado a uma redução de 11,0% na probabilidade de ansiedade (OR = 0,89; IC 95%: 0,84–0,95) e uma redução de 11,0% na probabilidade de depressão (OR = 0,89; IC 95%: 0,85–0,94). Para um incremento de 10,0%, essas reduções foram de 20,0% para ansiedade (OR = 0,80; IC 95%: 0,71–0,90) e 21,0% para depressão (OR = 0,79; IC 95%: 0,72–0,88).

Esses resultados indicam que a melhor saúde mental positiva e a maior autoeficácia estão associadas a menores níveis de ansiedade e depressão, enquanto pior saúde geral está associada a maiores níveis de ansiedade e depressão.

## DISCUSSÃO

Este estudo revelou uma elevada prevalência de sintomas de ansiedade e depressão entre estudantes de enfermagem, associada a fatores psicossociais como sobrecarga acadêmica, estresse, ausência de lazer e relações familiares conflituosas. A forte correlação entre ansiedade e depressão destaca a sobreposição desses transtornos e seu impacto negativo na saúde geral.

Os achados sugerem que os sintomas de ansiedade e depressão entre os estudantes estão relacionados a múltiplos fatores interligados, incluindo condições de vida, suporte social, saúde mental pregressa e dinâmica acadêmica. A magnitude das associações encontradas ressalta a necessidade de ações institucionais voltadas à promoção da saúde mental, com foco preventivo e assistencial, especialmente em grupos mais vulneráveis.

A percepção reduzida de autoeficácia entre estudantes com sintomas ansiosos e depressivos evidencia como tais condições fragilizam a confiança nas próprias capacidades, prejudicando o desempenho acadêmico e o bem-estar^(19)^. Assim, reforça-se a necessidade de intervenções voltadas à promoção da autoeficácia e à gestão do estresse acadêmico.

Embora a maioria dos participantes possuísse estabilidade financeira e residisse com a família, fatores tradicionalmente protetores, as elevadas prevalências de sofrimento psíquico indicam que esses elementos não foram suficientes para mitigar os efeitos negativos do contexto pandêmico. Ademais, o gênero feminino apresentou maior vulnerabilidade, conforme evidenciado em estudos prévios^(20,21)^, o que aponta a necessidade de políticas sensíveis às especificidades de gênero.

As condições de moradia também se mostraram relevantes: morar sozinho ou com amigos associou-se a maior prevalência de sintomas depressivos, indicando a solidão como fator de risco^(22)^. A presença de automutilação (26%) e ideação suicida (39,9%) entre os estudantes é alarmante, corroborando achados de outros estudos sobre o agravamento do sofrimento psíquico na pandemia^(7,23)^.

A correlação negativa entre autoeficácia e os sintomas de ansiedade e depressão reforça seu papel protetor. Estudantes com níveis mais elevados de autoeficácia tendem a apresentar menor sofrimento emocional, além de demonstrarem maior engajamento acadêmico, em contextos nos quais a saúde mental positiva se mostra um fator determinante^(19,24)^.

As associações estatisticamente significativas entre saúde mental positiva, autoeficácia e engajamento acadêmico^(24)^ demonstram que o fortalecimento de aspectos positivos é essencial, não apenas para o bem-estar individual, mas também para o sucesso acadêmico. Entretanto, o fato de 68,1% dos estudantes não terem acompanhamento psicológico revela a ausência de suporte institucional adequado, intensificando a vulnerabilidade psíquica.

O trabalho e o estudo simultâneos podem ter efeitos ambivalentes: enquanto alguns estudantes relatam que o ambiente profissional favorece a socialização e melhora a saúde mental^(25)^, para outros pode intensificar o estresse, especialmente quando há ausência de estratégias adaptativas.

O estresse acadêmico, quando mal gerido, pode desencadear o uso de substâncias como forma de alívio, prejudicando ainda mais a saúde mental^(26)^. Medidas preventivas, como a implementação de técnicas de gestão emocional, por exemplo, respiração diafragmática^(27)^, podem ser eficazes e de baixo custo.

Além disso, estudos indicam que intervenções estruturadas de promoção da autoeficácia, baseadas em estratégias de psicoeducação e saúde mental positiva, podem reduzir significativamente os níveis de estresse, ansiedade e depressão em estudantes universitários^(8,9)^. Esses programas, geralmente realizados em sessões semanais, demonstram impacto positivo mesmo em intervenções de curta duração^(9)^.

Os dados obtidos corroboram a necessidade de programas institucionais que promovam saúde mental positiva, fortalecendo autoeficácia e resiliência^(28)^. Estratégias como escrita reflexiva, apoio emocional e desenvolvimento de habilidades comunicativas são apontadas como eficazes no enfrentamento de estressores acadêmicos^(29)^.

Estudos recentes evidenciam que fatores como curso, gênero e ocupação influenciam a saúde mental, sendo estudantes de enfermagem particularmente vulneráveis^(26)^. Essa vulnerabilidade exige ações institucionais específicas, integrando o cuidado com a saúde mental ao processo de ensino-aprendizagem^(30)^.

Este estudo apresenta algumas limitações importantes. A amostra foi composta por estudantes de uma única instituição pública estadual, com amostragem não probabilística por conveniência, o que pode comprometer a representatividade, limitando a generalização dos achados para outras realidades regionais ou institucionais. A natureza transversal da pesquisa também impede estabelecer relações de causalidade entre as variáveis estudadas. Além disso, a coleta de dados online, embora necessária em função do distanciamento social, pode ter gerado viés de seleção, excluindo estudantes com menor acesso à internet ou menos engajados com as atividades acadêmicas.

Apesar dessas limitações, este estudo oferece contribuições relevantes ao conhecimento existente. Ao integrar variáveis pouco exploradas de forma conjunta, como saúde mental positiva, autoeficácia e sofrimento psíquico, os achados ampliam a compreensão dos fatores de proteção e risco entre estudantes de Enfermagem. As evidências obtidas podem subsidiar o desenvolvimento de programas institucionais focados na promoção do bem-estar, prevenção de transtornos emocionais e fortalecimento das competências socioemocionais na formação em saúde.

## CONCLUSÃO

O estudo evidenciou elevada prevalência de ansiedade e depressão entre estudantes de enfermagem, associadas a múltiplos fatores psicossociais e acadêmicos, como sobrecarga, estresse frequente, ausência de lazer e suporte psicológico limitado. A percepção de autoeficácia e a saúde mental positiva demonstraram papel protetor relevante, correlacionando-se negativamente com os sintomas de sofrimento psíquico e positivamente com o bem-estar e o engajamento acadêmico.

Os resultados apresentados oferecem evidências úteis para orientar práticas pedagógicas e políticas institucionais voltadas à saúde mental no ensino superior. Eles também apontam caminhos viáveis para intervenções psicoeducativas, ações de prevenção e fortalecimento do apoio emocional no ambiente acadêmico, especialmente quando articuladas a estratégias estruturadas que promovam ambientes acadêmicos mais saudáveis, com foco na redução de estressores, no fortalecimento das competências socioemocionais, na promoção da saúde mental positiva e na ampliação do acesso a suporte psicológico. A incorporação dessas ações ao projeto pedagógico dos cursos de Enfermagem pode favorecer não apenas a saúde dos estudantes, mas também sua formação ética, crítica e resiliente frente aos desafios da profissão.

Recomenda-se o desenvolvimento de novos estudos com amostras mais amplas, multicêntricas e delineamentos longitudinais, capazes de aprofundar a compreensão das relações causais entre saúde mental, autoeficácia e desempenho acadêmico, especialmente em contextos de elevada vulnerabilidade como no caso da pós-pandemia.

## Data Availability

Os dados que dão suporte aos resultados deste estudo estão disponíveis mediante solicitação ao autor correspondente, devido a requisitos de aprovação ética.

# Translation: Salud general, ansiedad y depresión: relación con la autoeficacia percibida y la salud mental positiva en estudiantes de enfermería*

**DOI:** 10.1590/1980-220X-REEUSP-2025-0260es

**Authors:** Eliel de Oliveira Bandeira, Daniele Alcalá Pompeo, Natália Sperli Geraldes Marin dos Santos Sasaki, Camila Garcel Pancote, Luiz Antônio Alves de Menezes-Júnior, Danilo de Miranda Alves, Letícia Palota Eid, Luciano Garcia Lourenção

**Affiliations:** 1 Universidade Federal do Rio Grande, Escola de Enfermagem, Rio Grande, RS, Brasil.; 2 Faculdade de Medicina de São José do Rio Preto, São José do Rio Preto, SP, Brasil.; 3 União das Faculdades dos Grandes Lagos, São José do Rio Preto, SP, Brasil.; 4 Universidade Federal de Ouro Preto, Escola de Nutrição, Ouro Preto, MG, Brasil.; 5 Universidade Federal de Jataí, Instituto de Ciências da Saúde, Jataí, GO, Brasil.

**Keywords:** Estudiantes de Enfermería, Salud Mental, Autoeficacia, Ansiedad, Depresión

## Abstract

**Objetivo:**

Analizar las condiciones de salud general, ansiedad y depresión de los estudiantes de enfermería y analizar su relación con la percepción de autoeficacia general y salud mental positiva durante la lucha contra la COVID-19.

**Método:**

Estudio transversal, descriptivo y correlacional, con 138 estudiantes de enfermería de una institución pública. Se aplicaron instrumentos para variables sociodemográficas, Escala Hospitalaria de Ansiedad y Depresión, Escala de Autoeficacia General, Cuestionario de Salud General y de Salud Mental Positiva. Los análisis incluyeron estadística descriptiva, correlaciones y regresión logística univariante y multivariante.

**Resultados:**

Un aumento del 5 % en la puntuación de Salud Mental Positiva se asoció con una reducción del 20,4 % en la probabilidad de ansiedad (OR = 0,83; IC del 95 %: 0,73–0,93) y del 26,6 % en la de depresión (OR = 0,79; IC 95 %: 0,70–0,89). Incrementos similares en el Cuestionario de Salud General se asociaron con mayores probabilidades de ansiedad (29 %) y depresión (18 %). Un aumento del 5 % en la percepción de autoeficacia general redujo en un 12,4 % la probabilidad de ambos resultados (ansiedad y depresión).

**Conclusión:**

La salud mental positiva y la autoeficacia fueron factores protectores, lo que refuerza la importancia de las acciones institucionales para promover el bienestar académico.

## INTRODUCCIÓN

La transición a la vida universitaria representa una etapa crítica en el desarrollo de los adultos jóvenes, marcada por cambios significativos en el modelo de enseñanza-aprendizaje, en las interacciones sociales y en la estructura emocional^(1)^. Para los estudiantes del área de la salud, como los de enfermería, esta adaptación implica además vivir situaciones emocionalmente exigentes y de gran responsabilidad, que pueden comprometer el bienestar psicológico y aumentar la vulnerabilidad al desarrollo de trastornos mentales, como la ansiedad y la depresión^(2)^.

Varios estudios indican que los estudiantes de enfermería presentan niveles elevados de sufrimiento psíquico en comparación con sus compañeros de otras carreras, debido a la extensa carga horaria, la intensidad de las actividades prácticas y el contacto precoz con situaciones de dolor, sufrimiento y muerte^(3)^. Estas condiciones hacen que este grupo sea especialmente susceptible a las enfermedades mentales, con implicaciones negativas en el rendimiento académico, el riesgo de abandono y la calidad de la formación profesional^(2,4)^.

La pandemia de covid-19 agravó esta situación al imponer medidas de aislamiento social y cambios abruptos en el proceso de enseñanza-aprendizaje, con la migración a la enseñanza a distancia y la suspensión de actividades prácticas esenciales para la formación en enfermería^(5)^. Las investigaciones han informado de un aumento significativo de los síntomas de ansiedad, estrés y depresión entre los estudiantes universitarios, especialmente los del área de la salud, que han pasado a ser reconocidos como un grupo prioritario para las acciones de promoción y atención de la salud mental^(6,7)^.

En este contexto, la autoeficacia, definida como la creencia en la capacidad de organizar y ejecutar acciones para alcanzar determinados objetivos, se ha destacado como un factor de protección para la salud mental de los estudiantes universitarios^(8)^. Las personas con altos niveles de autoeficacia tienden a afrontar los retos académicos como oportunidades de crecimiento, mostrando una mayor resiliencia emocional, una menor reactividad al estrés y una menor propensión a la ansiedad y la depresión^(9)^.

La salud mental positiva (SMP), por su parte, representa un enfoque centrado en el bienestar psicológico que va más allá de la mera ausencia de enfermedad mental. Implica aspectos como la satisfacción personal, el autocontrol, las habilidades relacionales, la autonomía y la realización personal^(10)^. La literatura ha enfatizado la importancia de su promoción desde los primeros momentos de la formación académica, como estrategia para fortalecer las competencias socioemocionales y prevenir el sufrimiento psíquico^(11)^.

A pesar de los avances en la producción científica sobre la salud mental de los universitarios, aún son escasos los estudios que analizan, de manera integrada, los vínculos entre la salud general, los síntomas emocionales (ansiedad y depresión), la autoeficacia percibida y la salud mental positiva, especialmente entre los estudiantes de enfermería en Brasil. Esta laguna se hace aún más evidente ante el impacto desproporcionado de la pandemia en este grupo y la necesidad de generar evidencias que fundamenten las acciones institucionales de acogida, prevención y promoción del bienestar universitario^(6)^.

Este estudio busca ampliar la literatura al examinar, de manera integrada, variables que tradicionalmente se analizan de forma aislada, como la salud mental positiva, la autoeficacia percibida, la ansiedad, la depresión y la salud general, contribuyendo a una comprensión más amplia de la salud mental de los estudiantes de enfermería. Se parte de la hipótesis de que los niveles más altos de salud mental positiva y autoeficacia están asociados con niveles más bajos de ansiedad y depresión, mientras que las peores condiciones de salud general están relacionadas con mayores riesgos de sufrimiento emocional.

Ante lo expuesto, el estudio tuvo como objetivo analizar las condiciones de salud general, ansiedad y depresión de los estudiantes de enfermería y analizar su relación con la percepción de autoeficacia general y salud mental positiva durante la lucha contra la COVID-19.

## MÉTODO

### Diseño del Estudio

Se trata de un estudio transversal, descriptivo y correlacional, realizado con estudiantes de enfermería de una institución de educación superior pública estatal del interior del estado de São Paulo, Brasil. El manuscrito se estructuró de acuerdo con las directrices de la herramienta STROBE (Strengthening the Reporting of Observational Studies in Epidemiology).

### Lugar del Estudio

El estudio se llevó a cabo en la Facultad de Medicina de São José do Rio Preto (Famerp), que ofrece cursos de licenciatura en Medicina, Enfermería y Psicología, además de cursos de posgrado Lato Sensu y Stricto Sensu.

La elección de la institución se debió a su relevancia en la formación de profesionales de la salud y al aumento de la demanda de servicios de apoyo psicológico entre sus estudiantes, observado especialmente durante la pandemia de covid-19.

### Población, Criterios de Selección Y Muestra

La población del estudio incluyó a 190 estudiantes matriculados en el curso de Enfermería en el año 2020. La identificación de los participantes se realizó mediante una lista proporcionada por la institución educativa. Los criterios de inclusión establecidos fueron: estar matriculado regularmente en el curso de licenciatura en enfermería de la institución; tener ≥ 18 años y estar presente en las fechas de recopilación de datos. Para el cálculo de la muestra, se consideró un nivel de confianza del 95 %, un margen de error del 5 % y una proporción esperada del 50 %, lo que dio como resultado una muestra mínima estimada de 128 estudiantes. Se adoptó un muestreo no probabilístico, por conveniencia, con la inclusión de todos los estudiantes que aceptaron participar y respondieron íntegramente a los instrumentos. Participaron en el estudio 138 estudiantes, lo que representa el 72,6 % de la población total, lo que garantiza una proporción adecuada para los objetivos del estudio.

### Recopilación de Datos

Para la recopilación de datos, se utilizaron cinco instrumentos autoaplicables: un cuestionario que contenía variables personales, sociales, académicas, hábitos de vida, además de preguntas sobre la presencia de estrés, sobrecarga y percepción del impacto de la pandemia en su vida; la Escala Hospitalaria de Ansiedad y Depresión (*Hospital Anxiety and Depression Scale* – HADS)^(12)^; la Escala de Autoeficacia General y Percibida^(13)^; el Cuestionario de Salud General (en portugués QSG-12)^(14)^; y el Cuestionario de Salud Mental Positiva (en portugués QSMP)^(15)^.

La HADS mide los síntomas sugestivos de ansiedad y depresión en entornos no psiquiátricos, basándose en la última semana del individuo. Cada ítem de la HADS contempla cuatro posibilidades de respuesta (0 a 3), con una puntuación máxima de 21 puntos para cada subescala^(12)^. Los estudiantes con una puntuación ≥ 9 en cada subescala se consideraron con ansiedad y/o depresión.

La Escala de Autoeficacia General y Percibida se compone de 10 ítems, con respuestas que varían de uno a cinco, donde 1 significa «totalmente en desacuerdo» y 5 «totalmente de acuerdo». Cada ítem mide las creencias que el individuo tiene sobre sus capacidades para alcanzar un objetivo, lidiar con una situación o realizar una tarea (Ejemplo: Siempre consigo resolver los problemas difíciles si lo intento con suficiente empeño; me resulta fácil aferrarme a mis objetivos y alcanzar mis metas). La puntuación se obtiene sumando los valores de los ítems, y una puntuación más alta indica una mayor percepción de autoeficacia, en un intervalo de 10 a 50^(13)^.

El QSG-12 corresponde a una versión abreviada del Cuestionario de Salud General, inicialmente elaborado con 60 ítems. La versión con 12 ítems es la más breve y la más utilizada para medir el bienestar psicológico, ya que las evidencias han demostrado que la reducción del número de ítems no compromete la fiabilidad de las respuestas. El QSG-12 se compone de 12 ítems que evalúan en qué medida la persona ha experimentado los síntomas descritos, con cuatro posibles respuestas. En los ítems que niegan la salud mental, las alternativas de respuesta pueden ser: (1) absolutamente no, (2) no más de lo habitual, (3) un poco más de lo habitual y (4) mucho más de lo habitual. En los ítems positivos, las respuestas varían entre: (1) más de lo habitual, (2) igual que siempre, (3) menos de lo habitual y (4) mucho menos de lo habitual. Se observa que los ítems negativos se han invertido y que la puntuación más baja indica un mejor nivel de bienestar^(14,16)^.

En Brasil, el QGS-12 ha demostrado evidencia de validez y precisión, además de consistencia interna, evaluada con un alfa de Cronbach superior a 0,80^(17)^. El Cuestionario de Salud Mental Positiva (QSMP) mide la salud mental positiva global mediante 39 afirmaciones sobre la forma de pensar, sentir y actuar de cada individuo^(15)^.

Estas afirmaciones se agruparon en seis factores: (F1) satisfacción personal, (F2) actitud positiva, (F3) autocontrol, (F4) autonomía, (F5) realización personal y resolución de problemas y (F6) habilidades de relación interpersonal. Las respuestas a cada ítem se presentan en una escala Likert de 4 puntos: (1) siempre o casi siempre, (2) la mayoría de las veces, (3) algunas veces, (4) casi nunca o nunca. Algunos ítems del instrumento se describen de forma positiva (n = 19) y otros de forma negativa (n = 20), en un intento de identificar posibles fallos en la cumplimentación, como la automatización en la marcación de los ítems. Las puntuaciones del QSMP varían de 39 (puntuación mínima) a 156 (puntuación máxima). Cuanto mayor es la puntuación obtenida, mayor es el nivel de salud mental positiva^(15)^. La versión en español fue validada en una muestra de 942 universitarios, con buenos resultados de consistencia interna (alfa de Cronbach = 0,92) y una estructura factorial similar a la del estudio original^(15)^.

Los datos se recopilaron en el segundo semestre de 2020. La invitación para participar en el estudio se envió a todos los estudiantes del curso de enfermería, al correo electrónico proporcionado en el momento de la inscripción y facilitado por la institución, y por mensaje de WhatsApp a los grupos de las clases de licenciatura. La invitación consistía en un video informativo que presentaba el título de la investigación, sus objetivos, los responsables de su realización y los instrumentos que debían responderse, además de destacar que la participación era voluntaria y que el estudio cumplía con las recomendaciones éticas para estudios con seres humanos.

Al aceptar participar en el estudio, los estudiantes hacían clic en un enlace disponible al final del video, que los dirigía al Término de Consentimiento Libre y Esclarecido (TCLE). Después de leerlo, los estudiantes tenían la opción de aceptar participar en el estudio. Tras confirmar su aceptación, los estudiantes tenían acceso a los instrumentos de recopilación de datos (cuestionario sociodemográfico, HADS, Escala de Autoeficacia General y Percibida, QSG-12 y QSMP, todos ellos disponibles a través de la plataforma Google Forms. El uso de esta herramienta fue esencial para la recopilación de datos, debido a que el estudio se llevó a cabo durante el período de aislamiento social derivado de la situación sanitaria generada por la pandemia de covid-19, en la que los estudiantes estaban alejados de las actividades in situ.

### Análisis y Tratamiento de Los Datos

El análisis estadístico se realizó con el software Stata® 15.0, adoptando un nivel de significación del 5 %. Inicialmente, se realizaron análisis descriptivos (frecuencias, medias y desviaciones estándar) y pruebas bivariadas (chi cuadrado de Pearson y t de Student) para comparar los resultados de ansiedad y depresión con variables sociodemográficas. La correlación entre las puntuaciones de HADS, QSG-12, Escala de Autoeficacia y QSMP se evaluó mediante el coeficiente de Spearman.

Se emplearon modelos de regresión logística univariante y multivariante para estimar la asociación entre las variables predictivas (autoeficacia, salud mental positiva y salud general) y los resultados (ansiedad y depresión), presentados en forma de odds ratios (OR) con un intervalo de confianza del 95 %. Aunque la regresión logística se recomienda tradicionalmente para resultados poco frecuentes, su aplicación en estudios transversales ha sido aceptada cuando el objetivo es identificar factores asociados a resultados dicotómicos, aunque sean prevalentes. Se optó por este modelo por su solidez y su amplio uso en investigaciones con un diseño similar.

Para las puntuaciones continuas de autoeficacia, salud mental positiva y salud general, se calcularon incrementos del 5 % y del 10 % en relación con los valores más bajos de las escalas, a partir de la siguiente fórmula: Porcentaje de incremento = [(Valor de la escala / Valor mínimo de la escala) – 1)] × 100. A partir de este porcentaje, se crearon variables que representaban incrementos del 5 % y del 10 %, que sustituyeron a los valores absolutos de las escalas. Este procedimiento permitió analizar cómo los aumentos del 5 % y del 10 % en los valores de las escalas, tomando como referencia el valor más bajo posible, se asociaban con los resultados de salud mental.

Para los modelos multivariados, se utilizó el procedimiento stepwise backward, con la inclusión inicial de variables con p < 0,20 en los análisis univariados y la permanencia en el modelo final basada en la significación estadística y la plausibilidad teórica. Este enfoque se recomienda para evitar la exclusión prematura de variables con potencial relevancia explicativa^(18)^.

Los modelos finales se ajustaron de la siguiente manera:

- Modelo para la ansiedad: ajustado por color de piel, práctica de ejercicio físico, percepción del estrés, sobrecarga académica y situación de vivienda;
- Modelo para la depresión: ajustado por relación familiar, satisfacción con el curso, color de piel, práctica de ejercicio físico, percepción del estrés, consumo de bebidas alcohólicas y práctica de actividades de ocio semanales.

### Aspectos Éticos

Este estudio forma parte del proyecto matricial «Salud mental positiva de los estudiantes de educación superior en salud», aprobado por el Comité de Ética en Investigación de la Facultad de Medicina de São José do Rio Preto con el dictamen n.º 4.095.190 y el Certificado de Presentación y Apreciación Ética n.º 31420620.8.0000. 5415. Todos los procedimientos siguieron las directrices de las Resoluciones n.º 466/2012 y n.º 510/2016 del Consejo Nacional de Salud. La recopilación se realizó en línea, con el consentimiento registrado mediante el Término de Consentimiento Libre e Informado electrónico, previamente presentado en un video explicativo.

## RESULTADOS

Participaron en el estudio 138 estudiantes de Enfermería, con predominio del sexo femenino (94.2%), edad media de 21.6 años (DP = 4.7) y que se declararon blancos (84.1%).

La mayoría no tenía pareja (65,2 %) y vivía con la familia (68,1 %), mientras que el 23,2 % residía con amigos y el 8,7 % vivía solo. En cuanto a la percepción de los recursos financieros, el 87 % consideraba que tenía recursos suficientes para sus necesidades.

En relación con la experiencia académica, el 90.6 % de los estudiantes informaron sentirse realizados con el curso, aunque el 89.1 % se sentían sobrecargados con las actividades de la licenciatura.

La mayoría no tenía un trabajo remunerado (80.4 %) y refería una sensación frecuente de estrés (86.2 %). La relación familiar fue calificada como armoniosa por el 83.3 % de los participantes. Solo el 42 % informó practicar actividad física con regularidad, y el 68.8 % afirmó tener actividades de ocio semanales.

El consumo frecuente de bebidas alcohólicas fue mencionado por el 14.5 % de los estudiantes, mientras que el tabaquismo y el consumo de drogas ilícitas fueron menos frecuentes, con un 5.1 % y un 3.6 %, respectivamente. El 31.9 % de los estudiantes mencionó haber recibido atención psicológica o psiquiátrica.

En cuanto a la salud mental, el 26 % de los participantes informaron haberse autolesionado en algún momento de su vida, el 10,1 % durante la licenciatura y el 15,9 % antes. El 39,9 % de los estudiantes mencionaron ideas suicidas, el 21 % durante la licenciatura y el 18,8 % antes de ingresar al curso.

La media de las puntuaciones de ansiedad y depresión fue de 10,72 (DE = 4,3) y 6,96 (DE = 3,93), respectivamente. Según los criterios adoptados, el 68,8 % de los estudiantes presentaban síntomas de ansiedad y el 37,0 %, de depresión.

El análisis bivariado evidenció asociaciones significativas entre los síntomas de ansiedad y las variables sociodemográficas y académicas (Tabla 1). Los estudiantes que vivían solos presentaron una mayor prevalencia de ansiedad (p = 0,020), lo que puede indicar una mayor vulnerabilidad emocional asociada al aislamiento social. La sobrecarga académica (p < 0,001), el estrés frecuente (p < 0,001) y la ausencia de ocio semanal (p = 0,001) también se destacaron como factores fuertemente asociados a la presencia de ansiedad. Además, las relaciones familiares conflictivas (p = 0,040), la ausencia de seguimiento psicológico o psiquiátrico (p = 0,008), los antecedentes de autolesión (p = 0,001) y las ideas suicidas (p < 0,001) antes y durante la licenciatura se asociaron significativamente con el resultado.

**Tabla 1:** Distribución de las características de los alumnos con ansiedad y depresión, según variables sociodemográficas y académicas – São José do Rio Preto, SP, Brasil, 2020. (n = 138).

Variables	Ansiedad n (%)	p-valor	Depresión n (%)	p-valor
**Color de piel**				
Blanco	83 (87,4)	0,114	47 (92,2)	**0,047**
No blanco	12 (12,6)		4 (7,8)	
**Situación de vida**				
Vive solo	12 (12,6)	**0,020**	7 (13,7)	0,134
Vive con la familia	65 (68,4)		30 (5,8)	
Vive con amigos	18 (19,0)		14 (27,5)	
**Te sientes feliz y realizado con tu carrera**				
No	10 (10,5)	0,509	9 (17,7)	**0,011**
Sí	85 (89,5)		42 (82,3)	
**Se siente sobrecargado de actividades de pregrado**				
No	3 (3,2)	**<0,001**	2 (3,9)	**0,045**
Sí	92 (96,8)		49 (96,1)	
**Trabaja remuneradamente**				
No	74 (77,9)	0,264	36 (70,6)	**0,026**
Sí	21 (22,1)		15 (29,4)	
**Se siente estresado con frecuencia**				
No	4 (4,2)	**<0,001**	1 (2,0)	**0,002**
Sí	91 (95,8)		50 (98,0)	
**Relación con la familia**				
Armoniosa	75 (78,9)	**0,040**	37 (72,6)	**0,009**
Conflictivo	20 (21,1)		14 (27,5)	
**Practica actividades de ocio semanalmente**				
No	38 (40,0)	**0,001**	27 (52,9)	**<0,001**
Sí	57 (60,0)		24 (47,1)	
**Asesoramiento psicológico o psiquiátrico**				
No	58 (61,1)	**0,008**	29 (56,9)	**0,030**
Sí	37 (38,9)		22 (43,1)	
**¿Alguna vez te has hecho daño a propósito para aliviar el sufrimiento?**				
No	62 (65,3)	**0,001**	34 (66,7)	0,138
Sí, durante la graduación	14 (14,7)		5 (9,8)	
Sí, antes de la graduación	19 (20,0)		12 (23,5)	
**Tuvo ideación suicida**				
No	44 (46,3)	**<0,001**	23 (45,1)	**0,017**
Sí, durante la graduación	28 (29,5)		16 (31,4)	
Sí, antes de la graduación	23 (24,2)		12 (23,5)	

En cuanto a la depresión, los resultados también revelaron un conjunto de factores asociados a su mayor prevalencia. Destaca la asociación con el color de piel blanco (p = 0,047), la falta de rendimiento en el curso (p = 0,011), el trabajo remunerado simultáneo a la carrera (p = 0,026), la sobrecarga académica (p = 0,045), el estrés (p = 0,002), las relaciones familiares conflictivas (p = 0,009), la falta de tiempo libre (p < 0,001), la falta de apoyo psicológico (p = 0,030) y la ideación suicida (p = 0,017). Aunque la automutilación no fue estadísticamente significativa en relación con la depresión, la frecuencia del comportamiento entre los estudiantes con síntomas depresivos fue significativa.

### Matriz de Correlaciones

Como se muestra en la Tabla 2, los resultados de la matriz de correlaciones revelaron asociaciones significativas entre las puntuaciones de las diferentes escalas evaluadas. La puntuación de ansiedad mostró una fuerte correlación positiva con la puntuación de depresión (r = 0,703; p < 0,001) y con la puntuación del Cuestionario de Salud General 12 (QSG-12) (r = 0,743; p < 0,001), lo que indica que mayores niveles de ansiedad se asocian con mayores niveles de depresión y peor salud general. Por otro lado, la puntuación de ansiedad mostró una correlación negativa significativa con la puntuación de la Escala de Autoeficacia General Percibida (r = –0,520; p < 0,001) y con la puntuación total del Cuestionario de Salud Mental Positiva (QSMP) (r = –0,216; p = 0,011), lo que sugiere que una mayor ansiedad se asocia con una menor autoeficacia y una peor salud mental positiva.

**Tabla 2:** Matriz de correlación entre las variables ansiedad, depresión, salud general, autoeficacia general y salud mental positiva en estudiantes de enfermería – São José do Rio Preto, SP, Brasil, 2020.

	Puntuación de ansiedad	Puntuación de depresión	Total SMP	Puntuación QSG12	Puntuación de autoeficacia
**Puntuación de ansiedad**	–	r: +0,703 p < 0,001	r: –0,216 p = 0,011	r: +0,743 p < 0,001	r: –0,520 p < 0,001
**Puntuación Depresión**	r: +0,703 p < 0,001	–	r: –0,185 p = 0,030	r: +0,723 p < 0,001	r: –0,630 p < 0,001
**SMP total**	r: –0,216 p = 0,011	r: –0,185 p = 0,030	–	r: –0,172 p = 0,440	r: +0,166 p = 0,520
**Puntuación QSG12**	r: +0,743 p < 0,001	r: +0,723 p < 0,001	r: –0,172 p = 0,440	–	r: –0,619 p < 0,001
**Puntuación de autoeficacia**	r: –0,520 p < 0,001	r: –0,630 p < 0,001	r: +0,166 p = 0,520	r: –0,619 p < 0,001	–

Prueba de correlación de Spearman.

La puntuación de depresión también mostró una correlación negativa significativa con la puntuación total de QSMP total (r = –0,185; p = 0,030) y la puntuación de la Escala de Autoeficacia General Percibida (r = –0,630; p < 0,001), y una correlación positiva con la puntuación de QSG-12 (r = 0,723; p < 0,001). Esto indica que una mayor depresión se asocia con una peor salud mental positiva, una menor autoeficacia y un peor estado de salud general.

Además, la puntuación total del QSMP mostró una correlación negativa, aunque no significativa, con la puntuación del QSG-12 (r = –0,172; p = 0,440) y una correlación positiva no significativa con la puntuación de la Escala de Autoeficacia General Percibida (r = 0,166; p = 0,520). Por último, la puntuación del GSF-12 mostró una correlación negativa significativa con la puntuación en la Escala de Autoeficacia General Percibida (r = –0,619; p < 0,001), lo que indica que un peor estado de salud general se asocia con una menor autoeficacia.

### Regresión Logística

Los resultados de las regresiones logísticas univariantes, que se muestran en la Tabla 3, indicaron que un aumento del 5% en la puntuación total del QSMP se asoció con una reducción del 17,0% en la probabilidad de ansiedad (OR = 0,83; IC 95%: 0,73–0,93) y una reducción del 21,0% en la probabilidad de depresión (OR = 0,79; IC 95%: 0,70–0,89). Para un aumento del 10,0%, estas reducciones fueron incluso mayores, con un 32,0% menos de probabilidad de ansiedad (OR = 0,68; IC 95%: 0,54–0,87) y un 38,0% menos de probabilidad de depresión (OR = 0,62; IC 95%: 0,49–0,80).

**Tabla 3:** Modelos de regresión logística univariante y multivariante para ansiedad y depresión en relación a las variables salud general, autoeficacia general y salud mental positiva en estudiantes de enfermería – São José do Rio Preto, SP, Brasil, 2020.

	Ansiedad		Depresión	
	Univariante	Multivariante^1^	Univariante	Multivariante^2^
**Total SMP**				
Aumento 5%	0,86 (0,78–0,94)	0,83 (0,73–0,93)	0,82 (0,75–0,91)	0,79 (0,70–0,89)
Aumento del 10	0,73 (0,61–0,89)	0,68 (0,54–0,87)	0,68 (0,56–0,82)	0,62 (0,49–0,80)
**Puntuación QSG12**				
Aumento 5%	1,27 (1,17–1,38)	1,29 (1,18–1,42)	1,19 (1,12–1,27)	1,18 (1,11–1,26)
Aumento del 10	1,62 (1,38–1,90)	1,67 (1,39–2,01)	1,42 (1,26–1,61)	1,39 (1,22–1,59)
**Puntuación de autoeficacia**				
Aumento 5%	0,92 (0,88–0,95)	0,89 (0,84–0,95)	0,89 (0,85–0,93)	0,89 (0,85–0,94)
Aumento del 10	0,83 (0,78–0,90)	0,80 (0,71–0,90)	0,79 (0,73–0,86)	0,79 (0,72–0,88)

^1^ Modelo para ansiedad ajustado por color de piel, ejercicio físico, estrés percibido, sobrecarga académica y situación de vivienda;

^2^ Modelo para depresión ajustado por relación familiar, rendimiento en el curso, color de piel, ejercicio físico, estrés percibido, consumo de bebidas alcohólicas y tiempo de ocio semanal.

En el caso del QSG-12, un aumento del 5,0% en la puntuación se asoció con un aumento del 29,0% en la probabilidad de ansiedad (OR = 1,29; IC 95%: 1,18–1,42) y un aumento del 18,0% en la probabilidad de depresión (OR = 1,18; IC 95%: 1,11–1,26). Para un aumento del 10,0%, estos incrementos fueron del 67,0% (OR = 1,67; IC 95%: 1,39–2,01) para la ansiedad y del 39,0% (OR = 1,39; IC 95%: 1,22–1,59) para la depresión.

La autoeficacia mostró una relación protectora en relación con la ansiedad y la depresión. Un aumento del 5,0% en la autoeficacia se asoció con una reducción del 11,0% en la probabilidad de ansiedad (OR = 0,89; IC 95%: 0,84–0,95) y una reducción del 11,0% en la probabilidad de depresión (OR = 0,89; IC 95%: 0,85–0,94). Para un aumento del 10,0%, estas reducciones fueron del 20% para la ansiedad (OR = 0,80; IC 95%: 0,71–0,90) y del 21,0% para la depresión (OR = 0,79; IC 95%: 0,72–0,88).

Estos resultados indican que una mejor salud mental positiva y una mayor autoeficacia se asocian con niveles más bajos de ansiedad y depresión, mientras que una peor salud general se asocia con niveles más altos de ansiedad y depresión.

## DISCUSIÓN

Este estudio reveló una alta prevalencia de síntomas de ansiedad y depresión entre los estudiantes de enfermería, asociada a factores psicosociales como la sobrecarga académica, el estrés, la falta de ocio y las relaciones familiares conflictivas. La fuerte correlación entre la ansiedad y la depresión pone de relieve la superposición de estos trastornos y su impacto negativo en la salud general.

Los hallazgos sugieren que los síntomas de ansiedad y depresión entre los estudiantes están relacionados con múltiples factores interrelacionados, incluyendo las condiciones de vida, el apoyo social, la salud mental previa y la dinámica académica. La magnitud de las asociaciones encontradas destaca la necesidad de acciones institucionales dirigidas a la promoción de la salud mental, con un enfoque preventivo y asistencial, especialmente en los grupos más vulnerables.

La reducida percepción de autoeficacia entre los estudiantes con síntomas de ansiedad y depresión pone de manifiesto cómo estas condiciones debilitan la confianza en sus propias capacidades, perjudicando el rendimiento académico y el bienestar^(19)^. Por lo tanto, se refuerza la necesidad de intervenciones dirigidas a promover la autoeficacia y la gestión del estrés académico.

Aunque la mayoría de los participantes gozaban de estabilidad financiera y residían con su familia, factores tradicionalmente protectores, la elevada prevalencia de sufrimiento psíquico indica que estos elementos no fueron suficientes para mitigar los efectos negativos del contexto pandémico. Además, el género femenino presentó una mayor vulnerabilidad, como se ha evidenciado en estudios previos^(20,21)^, lo que apunta a la necesidad de políticas sensibles a las especificidades de género.

Las condiciones de vivienda también resultaron relevantes: vivir solo o con amigos se asoció con una mayor prevalencia de síntomas depresivos, lo que indica que la soledad es un factor de riesgo^(22)^. La presencia de autolesiones (26 %) e ideas suicidas (39,9 %) entre los estudiantes es alarmante, lo que corrobora los hallazgos de otros estudios sobre el agravamiento del sufrimiento psíquico durante la pandemia^(7,23)^.

La correlación negativa entre la autoeficacia y los síntomas de ansiedad y depresión refuerza su papel protector. Los estudiantes con niveles más altos de autoeficacia tienden a presentar un menor sufrimiento emocional, además de demostrar un mayor compromiso académico, en contextos en los que la salud mental positiva se muestra como un factor determinante^(19,24)^.

Las asociaciones estadísticamente significativas entre la salud mental positiva, la autoeficacia y el compromiso académico^(24)^ demuestran que el fortalecimiento de los aspectos positivos es esencial, no solo para el bienestar individual, sino también para el éxito académico. Sin embargo, el hecho de que el 68,1 % de los estudiantes no reciba atención psicológica revela la ausencia de un apoyo institucional adecuado, lo que intensifica la vulnerabilidad psíquica.

El trabajo y el estudio simultáneos pueden tener efectos ambivalentes: mientras que algunos estudiantes informan que el entorno profesional favorece la socialización y mejora la salud mental^(25)^, para otros puede intensificar el estrés, especialmente cuando no existen estrategias de adaptación.

El estrés académico, cuando se gestiona mal, puede desencadenar el consumo de sustancias como forma de alivio, lo que perjudica aún más la salud mental^(26)^. Las medidas preventivas, como la implementación de técnicas de gestión emocional, por ejemplo, la respiración diafragmática^(27)^, pueden ser eficaces y de bajo costo.

Además, los estudios indican que las intervenciones estructuradas para promover la autoeficacia, basadas en estrategias de psicoeducación y salud mental positiva, pueden reducir significativamente los niveles de estrés, ansiedad y depresión en los estudiantes universitarios^(8,9)^. Estos programas, que suelen realizarse en sesiones semanales, demuestran un impacto positivo incluso en intervenciones de corta duración^(9)^.

Los datos obtenidos corroboran la necesidad de programas institucionales que promuevan la salud mental positiva, para fortalecer la autoeficacia y la resiliencia^(28)^. Estrategias como la escritura reflexiva, el apoyo emocional y el desarrollo de habilidades comunicativas se consideran eficaces para hacer frente a los factores estresantes académicos^(29)^.

Estudios recientes evidencian que factores como el curso, el género y la ocupación influyen en la salud mental, siendo los estudiantes de enfermería particularmente vulnerables^(26)^. Esta vulnerabilidad exige acciones institucionales específicas, integrando el cuidado de la salud mental en el proceso de enseñanza-aprendizaje^(30)^.

Este estudio presenta algunas limitaciones importantes. La muestra estaba compuesta por estudiantes de una única institución pública estatal, con un muestreo no probabilístico por conveniencia, lo que puede comprometer la representatividad y limitar la generalización de los resultados a otras realidades regionales o institucionales. La naturaleza transversal de la investigación también impide establecer relaciones de causalidad entre las variables estudiadas. Además, la recolección de datos en línea, aunque necesaria debido al distanciamiento social, puede haber generado un sesgo de selección, excluyendo a los estudiantes con menor acceso a Internet o menos comprometidos con las actividades académicas.

A pesar de estas limitaciones, este estudio ofrece contribuciones relevantes al conocimiento existente. Al integrar variables poco exploradas de forma conjunta, como la salud mental positiva, la autoeficacia y el sufrimiento psíquico, los hallazgos amplían la comprensión de los factores de protección y riesgo entre los estudiantes de Enfermería. Las evidencias obtenidas pueden contribuir al desarrollo de programas institucionales centrados en la promoción del bienestar, la prevención de trastornos emocionales y el fortalecimiento de las competencias socioemocionales en la formación en salud.

## CONCLUSIÓN

El estudio evidenció una alta prevalencia de ansiedad y depresión entre los estudiantes de enfermería, asociada a múltiples factores psicosociales y académicos, como la sobrecarga, el estrés frecuente, la falta de ocio y el apoyo psicológico limitado. La percepción de autoeficacia y la salud mental positiva demostraron tener un papel protector relevante, correlacionándose negativamente con los síntomas de sufrimiento psíquico y positivamente con el bienestar y el compromiso académico.

Los resultados presentados ofrecen evidencia útil para orientar las prácticas pedagógicas y las políticas institucionales orientadas a la salud mental en la educación superior. También señalan vías viables para intervenciones psicoeducativas, acciones de prevención y fortalecimiento del apoyo emocional en el entorno académico, especialmente cuando se articulan con estrategias estructuradas que promueven entornos académicos más saludables, centradas en la reducción de los factores estresantes, el fortalecimiento de las competencias socioemocionales, la promoción de la salud mental positiva y la ampliación del acceso al apoyo psicológico. La incorporación de estas acciones al proyecto pedagógico de los cursos de Enfermería puede favorecer no solo la salud de los estudiantes, sino también su formación ética, crítica y resiliente frente a los retos de la profesión.

Se recomienda el desarrollo de nuevos estudios con muestras más amplias, multicéntricas y diseños longitudinales, capaces de profundizar en la comprensión de las relaciones causales entre la salud mental, la autoeficacia y el rendimiento académico, especialmente en contextos de alta vulnerabilidad como en el caso de la post-pandemia.

## Data Availability

Los datos que respaldan los resultados de este estudio están disponibles previa solicitud al autor correspondiente, debido a requisitos de aprobación ética.
